# Influence of *Gliricidia sepium* living support on rhizosphere bacterial recruitment and fruit metabolomics in black pepper (*Piper nigrum*)

**DOI:** 10.1007/s11274-026-05278-1

**Published:** 2026-10-31

**Authors:** Valéria Alves Chaves, Oscar Victor Cardenas Alegria, Carlos Willian Dias Dantas, Victor Benedito Costa Ferreira, David Tavares Martins, Rhodin Clarentz Joseph, Laís Sousa Almeida, Sandro Duarte de Souza, Paulo Wender P. Gomes, Joyce Kelly R da Silva, Rommel Thiago Jucá Ramos

**Affiliations:** 1 Socioenvironmental and Water Resources Institute, Federal Rural University of the Amazon - UFRA, Belém, Pará Brazil; 2 Laboratory of Simulation and Computational Biology, High Performing Computer Center, Federal University of Pará - UFPA, Belém, Pará Brazil; 3 Laboratório de Bioprospecção e Inovação Tecnológica de Produtos Naturais da Amazônia, Federal University of Pará - UFPA, Belém, Pará Brazil; 4 Amazon Integrated Metabolomics Center, Institute of Exact and Natural Sciences, Federal University of Pará - UFPA, Belém, Brazil; 5 Institute of Biological Science, Federal University of Minas Gerais - UFMG, Belo Horizonte, Minas Gerais Brazil; 6 Laboratory of Bioinformatics and Genomics of Microorganisms, Institute of Biological Science, Federal University of Pará - UFPA, Belém, Pará Brazil

**Keywords:** Black pepper, Rhizosphere, Antimicrobial resistance (AMRs), Antimicrobial resistance gene (ARGs), Recruitment, *Actinobacteria*, Metabolomic profile

## Abstract

The ability of the rhizosphere to recruit beneficial microorganisms is central to the sustainable management of crops. Here, we investigated, through shotgun metagenomic sequencing and mass spectrometry techniques, the microbial composition, antimicrobial resistance (AMRs), and metabolomic profiles of *Piper nigrum* cultivated under two support systems: living *Gliricidia sepium* support (SL/RL) and dead wooden support (SD/RD). Bulk and rhizosphere soils were sequenced to assess taxonomic structure and ARGs, with emphasis on *Actinobacteria* due to their relevance in antibiotic production. *Actinobacteria* were highly represented across the samples (~ 35%). Dead-support rhizospheres exhibited a broader ARG repertoire and greater abundance of taxa known to include antibiotic-producing members, whereas living-support rhizospheres showed reduced ARG diversity. Metabolomic analysis further revealed support-dependent physiological responses in the host plant. PLS-DA distinguished the two cultivation systems, and three defense-related flavonoids, such as sakuranetin, isovitexin, and orientin, which were enriched in fruits of plants grown with dead support, indicating activation of stress – and defense – associated metabolic pathways. Their low abundance in living-support plants suggests reduced physiological stress, likely linked to improved nutrient cycling and rhizosphere stability provided by *G. sepium*. However, the volatile and total phenolic profile did not display significant chemical alterations in the samples. Together, these results suggest that support type influences both microbial functions and plant secondary metabolism. Dead support creates a more stressful ecological context that enriches antimicrobial resistance genes ARGs and defense metabolites, whereas living support promotes a more balanced soil-plant-microbe environment, supporting the use of *G. sepium* as a sustainable and biologically advantageous cultivation strategy for black pepper.

**Supplementary Information:**

The online version contains supplementary material available at https://doi.org/10.1007/s11274-026-05278-1.

## Introduction

Brazil is the second-largest producer and exporter (15% of global exports) of black pepper (*Piper nigrum L.*) in the world, ranking behind only Vietnam (Sociedade Nacional de Agricultura, 2023). Black pepper is marketed in domestic and international markets for food purposes, and its byproducts, such as piperine and oleoresin, are used by the cosmetic and pharmaceutical industries. In Brazil, the states of Espírito Santo, Pará, and Bahia stand out as the largest producers of black pepper, accounting for the production of 125 thousand tons of black pepper, approximately 99% of national production (IBGE 2023). This crop faces obstacles related to diseases caused by pathogens, including *Phytophthora capsici* and *Fusarium* spp., fungi that cause root rot and are among the main agents responsible for losses in black pepper cultivation. Despite this, plants possess several natural mechanisms of resistance to stress, including the ability to associate with microorganisms that are beneficial to their growth (Queiroga et al. 2023; Chien and Gachomo, 2023). Black pepper is a climbing plant; therefore, supports are used in its cultivation, which can be of two types: dead support, in which wooden supports are used; and living support, in which intercropping with another plant is carried out. However, the use of dead support results in the unregulated exploitation of native timber from the Amazon region, especially hardwood species such as acapu (*Vouacapoua americana*) and jarana (*Eicheilera jarana*), which are commonly used for this purpose, contributing to the deforestation in the Amazon region. In the cultivation with living support, or shaded cultivation, *Gliricidia* plants (*Gliricidia sepium*), a leguminous species, are mainly used due to their ease of availability (Kill et al., 2005; Araújo et al. 2024). In addition to preserving vegetation, the use of living support requires only half of the fertilizer dose recommended for cultivation with dead support (Embrapa, 2004). For large-scale production, in order to protect crops from pests that affect productivity, agricultural inputs such as biocides with potential antimicrobial effects are used (Pignati et al. 2017). In addition to requiring high investments, the use of chemical pesticides also represents a threat to soil health and groundwater (Zheng et al. 2022). It is also noteworthy that the extensive use of antimicrobials and biocides in agriculture may lead to the selection of resistant organisms through adaptation via the acquisition of resistance genes whose mechanisms can be shared (Rad et al. 2022). Therefore, the search for more sustainable alternatives is fundamental for the maintenance and improvement of agricultural productivity (Rizvi et al. 2022). In this context, approaches that explore the potential use of microorganisms in agriculture have gained popularity. Among the main targets are rhizosphere bacteria with plant growth-promoting potential (PGPR) and bacteria that produce antimicrobial compounds that may act as biocontrol agents against phytopathogens, such as *Actinobacteria* (Rizvi et al., 2022). The phylum *Actinobacteria* is a group of interest due to its ability to produce a wide variety of compounds of commercial relevance. It is estimated that this group is associated with the production of more than 10,000 antibiotics (Karthik 2022). Among the main classes are macrolides (such as oligomycin and polyenes), daunomycin, cyclopoly-lactones (non-actin), aminoglycosides, streptomycin, polyethers (nigericin), cyclopoly-lactones, quinoxaline peptides, and actinomycins. The genus *Streptomyces*, belonging to this phylum, is responsible for the production of more than 80% of antibiotics derived from *Actinobacteria*; additionally, this genus also stands out for the production of compounds with antifungal activity (Karthik 2022; Syiemiong et al. 2022). These bacteria have been studied for their potential as biocontrol agents against phytopathogens. Moreover, some strains have been reported in the literature as having potential activity against some of the main pathogens of *Piper nigrum*, such as the fungi that cause root rot (Trindade et al. 2021).

The rhizosphere recruits microorganisms from the soil that have the potential to benefit the plant, and can have a direct impact on its growth by facilitating the acquisition of nutrients and by the modulation of phytohormones or indirectly through the inhibition of plant pathogens, either via competition or through the production of antimicrobial compounds (Olanrewaju et al. 2017). However, studies investigating how this microbial recruitment influences the selection and maintenance of antimicrobial resistance genes in the rhizosphere microbiome are still scarce.

In this context, actinobacteria represent a group of particular interest, as they are recognized for producing a wide variety of bioactive metabolites, including antibiotics and antifungal compounds, that can contribute to the biocontrol of phytopathogens and potentially shape the soil resistome. Thus, this study aims to evaluate the influence of *Gliricidia sepium* supports (living supports) versus stake supports (dead supports) on the diversity and composition of the rhizosphere microbiota, with an emphasis on the recruitment of *actinobacteria* and the characterization of antimicrobial resistance genes present in the samples using shotgun metagenomics techniques, as well as to characterize the metabolomic composition of *Piper nigrum* fruits.

## Methods

### Sample collection

Soil samples were collected in May 2022 from the experimental cultivation area of TROPOC – Produtos Tropicais de Castanhal, located in Castanhal, Pará, Brazil (1°17’36.9” S; 47°49’34.1” W). Sampling was conducted during the organic cultivation phase and included two plots of black pepper (*Piper nigrum L. cv. Bragantina*), both three years old. The plots were located close to each other due to the small size of the experimental area and were subjected to the same agronomic management practices, differing only in their support system.

One plot was cultivated with a living support (SL and RL samples), specifically *Gliricidia sepium*, whereas the other used a dead support (SD and RD samples) consisting of wooden stakes. For each cultivation system, two types of soil samples were collected: bulk soil and rhizosphere soil. Samples were collected as three independent biological replicates. For each experimental group (living support and dead support), three independent plants were selected in their respective cultivation plots using a transect sampling design. Each plant was considered a sampling unit and an independent biological replicate, and no subsamples were used to represent the biological replicates.

For each replicate, a soil sampler (probe-type) was inserted to a depth of 15–30 cm, and the procedure was repeated five times around the plant stem at a distance of 20 cm. The resulting soil material was pooled into a single container. The plant roots and soil samples were then separated and placed in 50-mL Falcon tubes. The samples were immediately stored in liquid nitrogen and transported to the laboratory for subsequent processing. Fruits were collected from the same sampling points used for soil and rhizosphere sampling.

### DNA extraction and sequencing

The bulk soil samples collected from different plants were preserved in liquid nitrogen. Initially, they were fragmented into 2 mL Eppendorf tubes and stored at −20 °C. One of these aliquots was used for the extraction of genetic material, following the manufacturer’s protocol for the DNeasy PowerSoil Pro Kit (Qiagen).

For the rhizosphere samples, 30 mL of buffer solution (0.75% KH₂PO₄, 0.95% K₂HPO₄, and 1% Triton X-100 in ddH₂O, filter-sterilized through a 0.2 μm filter) was added to the tube containing the root with adhered soil. The samples were then shaken for 30 min at 200 rpm. The resulting suspension was centrifuged and concentrated into 2 mL Eppendorf tubes for subsequent DNA extraction.

The quality of the extracted genetic material was assessed by horizontal gel electrophoresis, and spectrophotometric absorbance measurements were used to determine concentration and purity indices. Only extractions meeting quality criteria A₂₆₀/A₂₈₀ (1.8–2.0) were used for further analyses.

Shotgun metagenomic sequencing was performed to explore the diversity and genotype of the collected samples. Sequencing library preparation was performed using the Nextera XT DNA Library Preparation Kit (Illumina) to generate 2 × 150 bp paired-end reads on the Illumina NextSeq 500/550 High Output platform.

### Diversity analysis

Raw data were filtered using the Trimmomatic software (Bolger et al. 2014) to remove reads and/or read ends with a Phred quality score below 20, using a 4-base sliding window. Only reads longer than 49 base pairs after filtering were retained.

Taxonomic assignment of the sequences was performed using Kraken2 (Wood et al. 2019), with the “PlusPFP” database. Taxonomic abundance tables were generated using the Pavian platform (Breitwieser and Salzberg, 2020). Alpha diversity was calculated using the Shannon index with the AlphaDiversity module from the scikit-bio (skbio) Python library. Beta diversity was assessed through Analysis of Similarities (ANOSIM) and Non-metric Multidimensional Scaling (NMDS) using the MicrobiomeAnalyst platform (Lu et al. 2023).

For bacterial community composition analyses, data were first normalized using the MinMaxScaler from Python’s Scikit-learn (sklearn) library. Then, extreme outliers were removed using the IForest algorithm from the Alibi-detect library (v0.12.0). Graphs were generated using Orange Data Mining (v3.38.1) with the K-means clustering algorithm. To improve visualization, 50 clusters were selected, and both horizontal and vertical clustering were applied. From this cleaned dataset, a filtered table was generated containing only members of the phylum *Actinomycetota*. To improve visualization of bacterial families, a filter was applied using the interquartile range (IQR), retaining values between the median and the upper limit (IQR + 3).

### Antimicrobial resistance gene analysis

The analysis of antimicrobial resistance genes (ARGs) was performed using sequencing reads with the CARD tool (Alcock et al. 2023) and the WildCARD database (version 3.2.6). Abundance normalization of resistance genes was conducted as described by Inda-Diaz et al. (2023), considering only those genes with a mean coverage percentage greater than 75%. Fisher’s alpha diversity index was calculated using a custom Python script utilizing the skbio.diversity library.

### Extraction and analysis of volatile compounds from black pepper fruits

Black pepper fruits were collected and dried in a forced-air oven at 40 °C for 48 h. The dry material (100 g) was pulverized and submitted to hydrodistillation using a Clevenger-type apparatus (3 h). The oils were dried over anhydrous sodium sulfate essential oils were obtained from. The essential oil was analyzed using gas chromatography–mass spectrometry (GC-MS) as previously described (Trindade et al. 2021) Shimadzu QP2010 plus instrument; Rtx-5MS capillary column (30 m × 0.25 mm × 0.25 mm film thickness); temperature ramp of 60–240 °C at a rate of 3 °C/min; injector temperature of 200 °C; He carrier gas with 1.2 mL/min flow rate; split mode injection (20:1 split ratio); MS ionization voltage = 70 eV; ion source and detector temperature = 200 °C. Compound identification was achieved by comparison of the retention indices, determined using a homologous series of n-alkanes (C8–C32, Sigma–Aldrich, St. Louis, MO USA) (Van Den Dool and Kratz 1963) and the mass spectral fragmentation patterns with those reported in the databases (Adams 2012; NIST 2025; Luigi 2011).

### Extracts obtention

The extraction was performed using 2.0 g of dried fruits of *P. nigrum* by percolation (96 h) with 25 mL of methanol. Every 48 h, the samples were placed on ultrasound for 10 min. At the end of 96 h, the solvent was evaporated (Barata et al. 2021).

### Total phenolics determination

Extracts were dissolved in methanol at a concentration of 20 mg·mL^− 1^ and subsequently diluted with distilled water. A 500 µL aliquot of extract was combined with 250 µL of Folin–Ciocalteu reagent (1 N) and 1250 µL of sodium carbonate (75 g·L^− 1^). The reaction mixture was incubated in the dark for 30 min. Absorbance was measured at 760 nm using a UV-visible spectrophotometer (KASVI, K37-UVVIS). A calibration curve was generated with gallic acid (Sigma Aldrich, St. Louis, MO, USA) at concentrations ranging from 0.5 to 10.0 mg·L^− 1^. Total phenolic content was reported as gallic acid equivalents (GAE) in milligrams per gram of extract (mg GAE·g^− 1^) (Barata et al. 2021).

### Liquid chromatography-tandem mass spectrometry analysis

The liquid chromatography-tandem mass spectrometry (LC-MS/MS) analysis was carried out using a Xevo G2-S QTof high-resolution mass spectrometer (Waters Corp., Milford, MA, USA) equipped with a Lockspray source. Leucine-enkephalin served as the reference compound to ensure accurate mass measurements. System control and data acquisition were performed using MassLynx 4.1 software. Aliquots containing 2 µL of each extract, prepared at a concentration of 1000 ppm, were injected and separated using a BEH C18 column (Waters Corp.; 50 mm × 2.1 mm; 1.7 μm particle size) maintained at 40 °C. The mobile phase consisted of ultrapure water (solvent A) and acetonitrile (solvent B), with a flow rate of 300 µL/min. The chromatographic run lasted 15 min, following this gradient: 0–10 min (linear increase from 5% to 95% B), 10.01–12.0 min (column washing), 12.01–14.0 min (linear decrease from 95% to 5% B), and 14.01–15.0 min (maintained at 5% B). Data acquisition was performed in negative ionization (NI) mode within a mass range of m/z 50–1500. A Data-Dependent Acquisition (DDA) approach was used, applying a centroid format and selecting the five most intense ions (Top 5 Experiment). The collision energy was varied between 15 and 45 eV. The scan time was set to 0.1 s, with a charge state of + 2, a tolerance window of ± 0.2 Da, and a peak extraction tolerance of 2 Da. Deisotoping parameters included a tolerance of ± 3 Da and an extraction tolerance of 6 Da. The source and desolvation temperatures were maintained at 150 °C and 300 °C, respectively. The cone gas and desolvation gas flow rates were adjusted to 50 L/h and 800 L/h, respectively. The capillary voltage was set at 3.0 kV, and the cone voltage was 40 V.

Mass spectrometry data files obtained from the Xevo G2-S QTof instrument for all extracts were converted to the mzXML format using MSConvert software (ProteoWizard, Palo Alto, CA, USA) (Chambers et al. 2012). Data processing was carried out with MZmine version 4.0 (Schmid et al. 2023). Precursor and fragment intensity thresholds were set at 1.0 × 10³ (MS1) and 8.0 × 10¹ (MS^2^), respectively. The total ion chromatograms (TICs) were generated using the ADAP algorithm. Retention times were considered within a range of 0.02 to 10.00 min, with a minimum group size of 3 and an intensity threshold of 1.0 × 10³. The maximum and minimum intensity values were established at 3.0 × 10⁴, and the scanner accuracy was configured at 0.002 *m/z* or 10 ppm. Chromatogram deconvolution was performed using the Local Minimum Resolver algorithm, applying an 80% chromatographic threshold. The minimum search interval for retention time/mobility was 0.050, with a precursor tolerance of 0.002 *m/z* or 10 ppm. Noise threshold settings were 15, while the absolute minimum height was 1.0 × 10⁴. The peak-to-peak ratio was set to 1.7, the peak duration ranged from 0.10 to 1.00, and the wavelength range (RT) was 0.05 to 0.15. Isotope detection was based on a peak window tolerance of 0.002 *m/z* or 10 ppm, a retention time tolerance of 0.2 min, and a maximum charge of 2. Peak alignment was conducted with an *m/z* tolerance of 3:1 per retention time and a retention time tolerance of 0.2 min. After alignment, the resulting data were filtered to eliminate duplicates and entries without an associated MS2 spectrum. The processed files were then exported in mgf and CSV formats for further analysis on the GNPS platform (Wang et al. 2016). Metabolite annotations were assigned by comparison of experimental MS/MS spectra against reference spectra available in the GNPS spectral libraries. Spectral matches were evaluated based on precursor mass accuracy, fragmentation pattern, number of matched fragment ions, cosine similarity, and literature data. Annotation confidence was assigned according to the Metabolomics Standards Initiative (MSI), with compounds classified as putatively annotated compounds (MSI level 2) (Fiehn et al. 2007).

### Statistical analysis

Data normality was assessed using the Shapiro–Wilk test. Statistical analyses included Kruskal–Wallis tests and independent Mann-Whitney. All analyses were performed using custom Python scripts based on the SciPy.stats and scikit-posthocs libraries.

For the evaluation of *Actinobacteria* species presence, the criterion was the detection of the bacterium in all three replicates of the group. Because samples corresponded to independent biological replicates (individual plants) rather than technical replicates, some degree of inter-replicate variation in resistance gene detection was expected a priori. Furthermore, as the experimental system was not subjected to anthropogenic antimicrobial or pesticide inputs, resistance genes were expected to occur at generally low abundance and potentially patchy distribution across individual plants, reflecting incidental carriage by rare or unevenly distributed microbial taxa rather than a consistently selected trait. Accordingly, gene presence within a group was defined using a comparatively permissive replicate criterion (≥ 2 aligned reads summed across the three replicates), while a stricter, conservative coverage threshold (> 75% of the reference gene length) was applied to minimize misidentification. ARGs were further evaluated through hierarchical clustering using a similarity matrix with the Morisita index via the Orange Data Mining tool (v3.38.1).

Graphs in this study were generated using Python (v3.11.11) scripts with the Upsetplot, Seaborn, Pandas, NumPy, and Matplotlib.pyplot libraries. Principal Component Analysis (PCA) was conducted using Matplotlib and Scikit-learn, with data standardization performed using StandardScaler from the Scikit-learn library.

For mass spectrometry data, statistical analyses were performed in the Jupyter Notebook. Global metabolic variation across sample types was assessed using Partial Least Squares-Discriminant Analysis (PLS-DA). Categorical variables were numerically encoded, and two components were extracted, containing the 50 top Variable Importance in Projection (VIP) metabolites, with explained variance calculated manually from centered data. Hierarchical clustering was conducted with seaborn’s clustermap, applying a custom diverging colormap to highlight metabolite intensity patterns. Both row and column clustering were used to identify co-regulated metabolite groups, and key discriminatory metabolites were annotated using automated label formatting and custom markers.

## Results

### Bacterial diversity and composition

#### Metagenomic data overview

Sequencing generated a total of 278.8 million paired-end reads across the 12 samples, with each sample producing between 4.0 and 49.5 million reads. The paired replicates from RD and RL generated between 4.0 and 5.9 million reads, totaling approximately 30 million reads corresponding to the rhizosphere samples. In contrast, SL and SD showed a substantial increase in sequencing depth, ranging from 125.9 to 132 million read pairs across the soil samples.

Sequencing quality was high across all samples, with mean Phred scores ranging from 35.2 to 35.8. GC content varied between 61% and 62%. Additionally, more than 99.78% of reads had a quality score ≥Q30, indicating a high proportion of high-quality bases. Differences in sequencing depth among samples were taken into account in the subsequent analyses, and the data were normalized as described in the Methods section.

#### Bacterial diversity and composition

The Kraken2 results showed that the identified organisms belonged predominantly to the domain Bacteria, accounting for an average of ~ 97% of the sample microbiomes.

Thus, taxonomic identification of up to 9,632 and 10,019 bacteria was performed in rhizospheric soil and bulk soil samples, respectively. The Shannon diversity index differed significantly the four groups according to the Kruskal–Wallis test for both bacteria (H = 8.74, *p* = 0.033) and *Actinomycetota* (H = 8.4359, *p* = 0.0378). However, none of the pairwise comparisons remained statistically significant after Bonferroni correction in Dunn’s *post-hoc* test, with the smallest adjusted *p-value* observed for the RL versus SD comparison (*p* = 0.076). This discrepancy likely reflects, at least in part, the limited statistical power associated with three biological replicates per group and the conservative adjustment for multiple comparisons.

In this way, the microbial diversity of species corresponding to the bacterial phylum *Actinobacteria* was higher in bulk soil, with values of up to 7.05, and in rhizospheric soils up to 6.99, and it was possible to observe a reduction in bacterial diversity between bulk soil and the rhizosphere in all samples (Table 1).

**Table 1:** Values of the total Shannon alpha diversity index for the phylum *Actinomycetota* in rhizosphere and bulk soil samples from the two support systems. “a” indicates no statistically significant differences detected

Sample	Type	Shannon Index (± standard deviation)	Shannon Actinomycetota (± standard deviation)
SD	Bulk soil with support	7.95 ± 0.013a	7.04 ± 0.011a
RD	Rhizosphere soil with support	7.84 ± 0.042a	6.98 ± 0.029a
SL	Bulk soil with living support of *Gliricídia sepium*	7.94 ± 0.020a	7.05 ± 0.015a
RL	Rhizosphere soil with living support of *Gliricídia sepium*	7.83 ± 0.030a	6.99 ± 0.002a

**Fig. 1:**
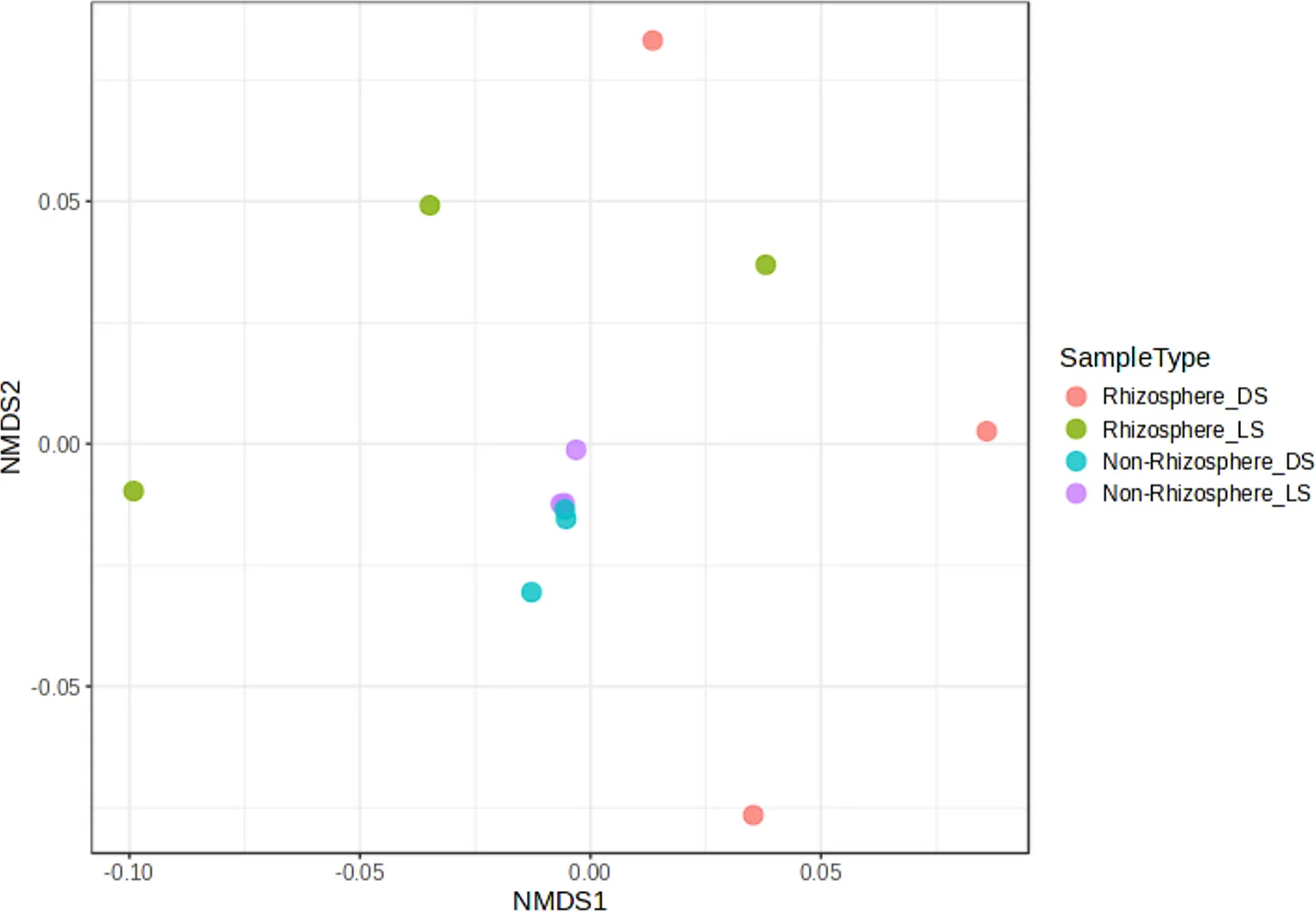
Beta-diversity analysis. NMDS ordination based on Bray-Curtis dissimilarity, with statistical significance assessed using ANOSIM

The beta-diversity index (NMDS Stress = 0.14168; ANOSIM (*R* = 0.49691; *p-value* < 0.005) (Fig. 1) indicates that the groups are moderately separated and that this separation is statistically significant. Based on the NMDS representation, the rhizosphere samples appear to be the factor most associated with this separation. The greater similarity between replicates within groups compared to the dissimilarity between groups indicates that there is a structure associated with the different types of sample treatments.

**Fig. 2:**
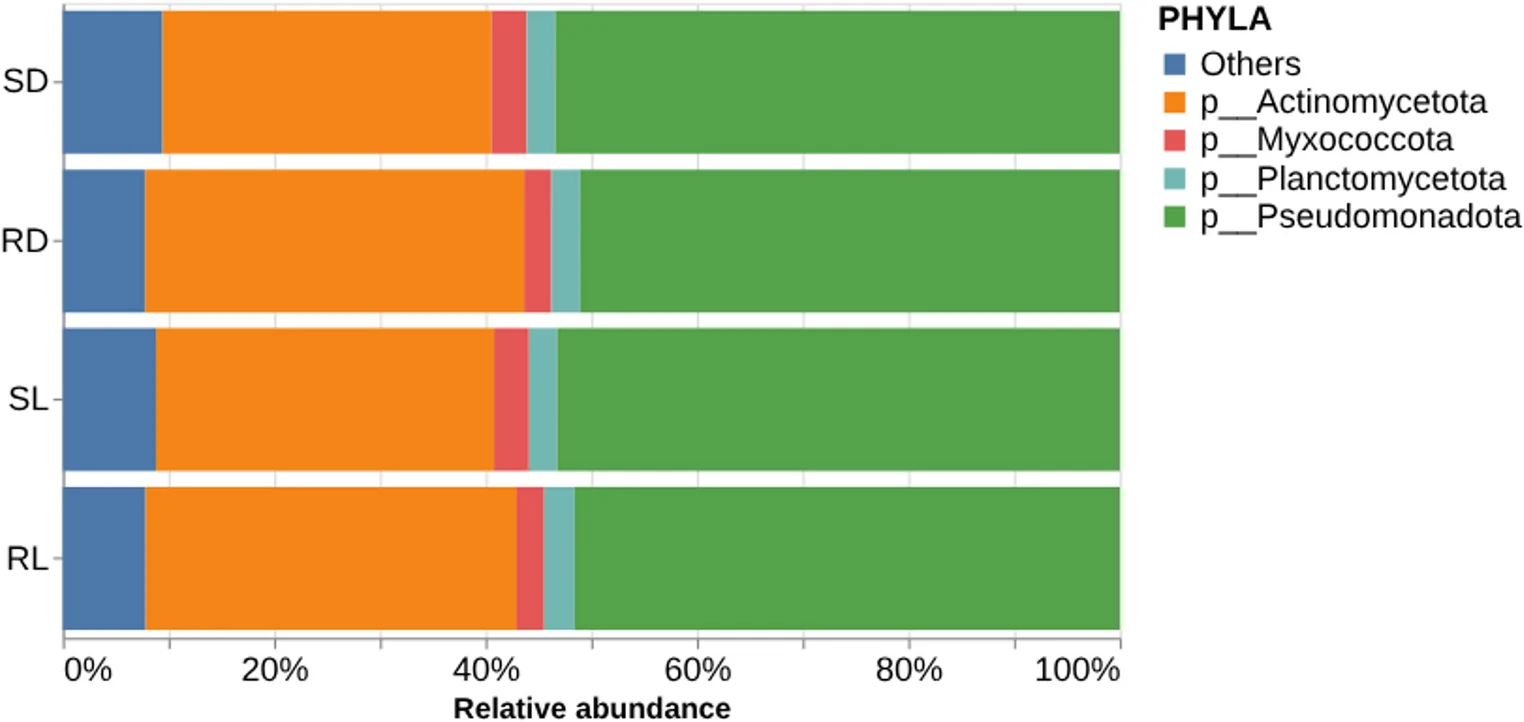
Relative abundance of bacterial phyla across bulk soil and rhizosphere samples under living and dead support systems. Bars represent the proportional composition of the main bacterial phyla, with less abundant taxa grouped as “Others.”

Among the bacterial phyla identified, the highest prevalence was observed for the phyla *Pseudomonadota* (~ 50%) and *Actinomycetota* (~ 35%), respectively (Fig. 2). These identified phyla represent more than 80% of the sample composition.

**Fig. 3:**
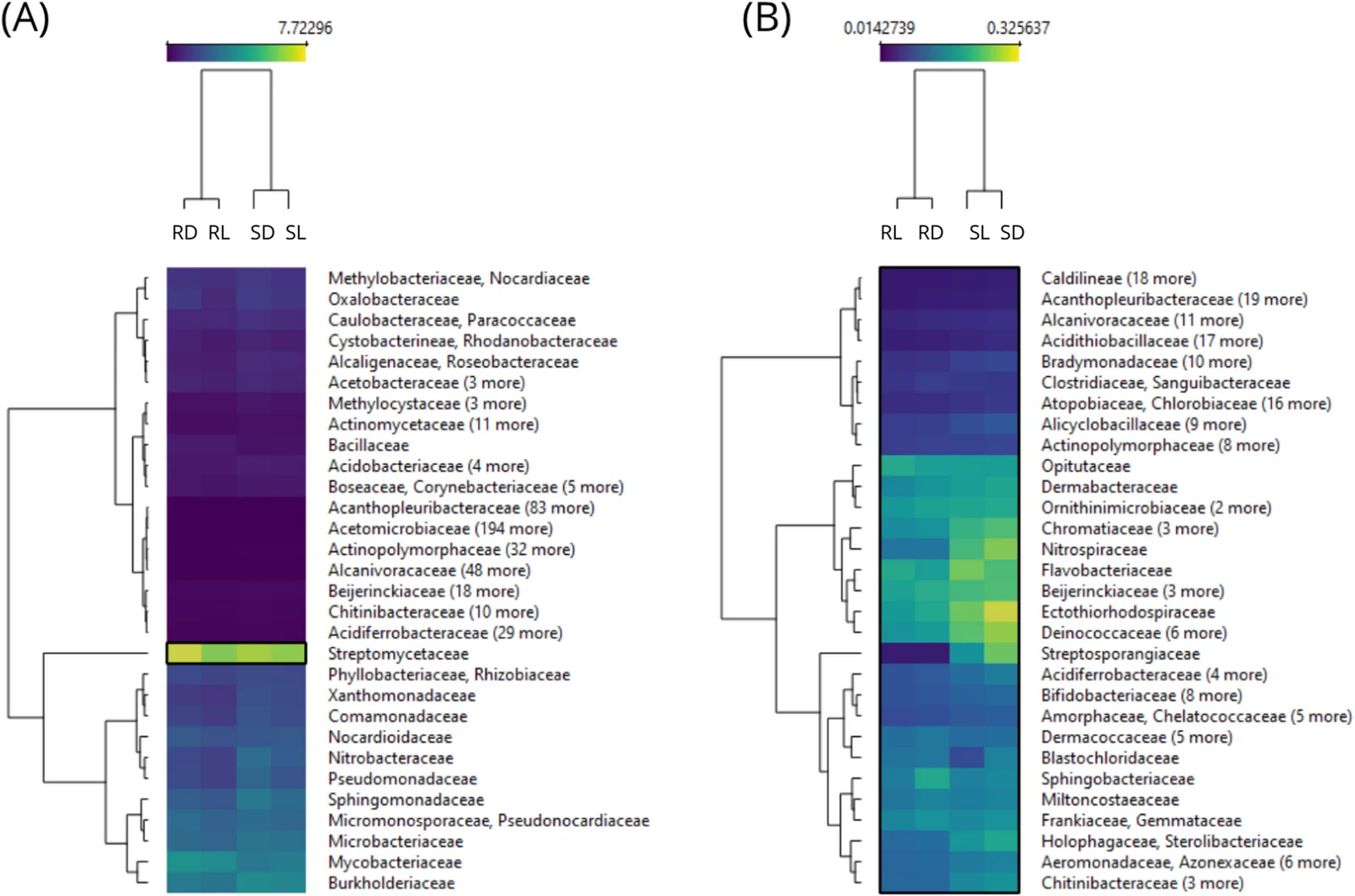
Heatmap of bacterial composition in the samples using K-means clustering. (**A**) after outlier filtering with Alibi-detect, and (**B**) after filtering extreme values by selecting the data range between the median and the upper limit (IQR + 3)

The bacterial composition was observed at the family level (Fig. 3). *Streptomycetaceae* stands out as the most abundant family in all samples, with higher incidence in RD. It is noteworthy that bulk soil samples cluster together, as do rhizospheric soil samples, indicating that there are more similarities related to the type of sample (bulk soil or rhizosphere) than to the support system.

In addition, other bacteria are prevalent in the samples, corresponding to the phyla *Actinomycetota* and *Pseudomonadota*. The abundance of Actinobacteria showed a similar distribution among samples, with the exception of the family *Mycobacteriaceae*, whose prevalence occurred in rhizospheric soil (RD and RL). Regarding the distribution of *Proteobacteria*, they were consistently more abundant in SD (followed by RD, SL, and RL, respectively). Among the Actinobacteria, the genus *Streptomyces* was the most abundant in all samples, especially in RD.

**Fig. 4:**
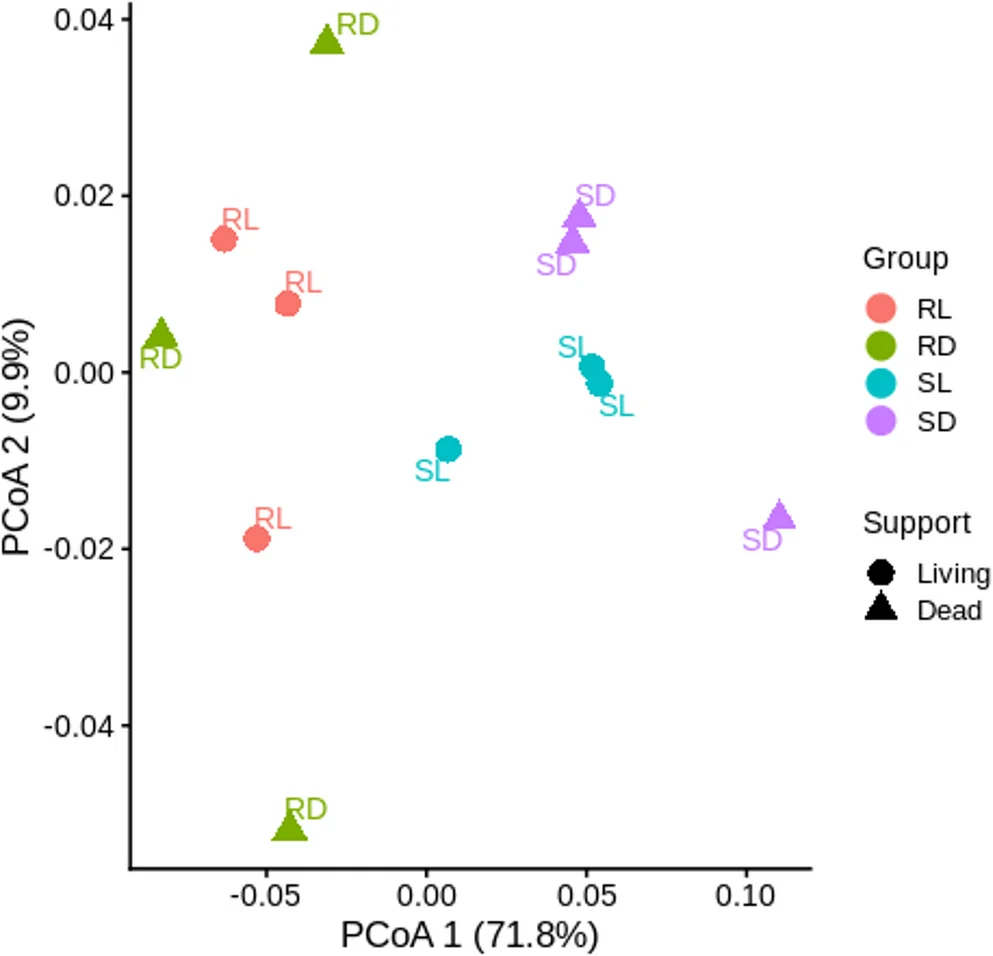
Principal Coordinates Analysis (PCoA) based on bray-curtis dissimilarity matrix. The plot illustrates the beta diversity distribution at the genus level, structured according to the PERMANOVA analysis. Axis 1 (PCoA 1) accounts for 71.8% of the total variation, driven primarily by sample type, while axis 2 (PCoA 2) accounts for 9.9%

The PCoA analysis (Fig. 4) based on the Bray–Curtis distance matrix used in the PERMANOVA (Supplementary Material – Table I) reinforces the separation between the bulk soil and rhizosphere groups, suggesting an important role of rhizosphere recruitment in bacterial community composition. Consistently, the factorial PERMANOVA identified sample type (bulk soil/rhizosphere) as the factor that best explains community variation, whereas support had no significant effect. The pairwise PERMANOVA analysis (Supplementary Material – Table II) did not detect statistically significant differences among the groups after corrections for multiple comparisons. Although some pairwise comparisons showed relatively high R² values, indicating substantial observed differences in community composition, the interpretation of this result needs to consider the statistical limitations related to the number of replicates per group.

### Profiles of actinobacteria species

**Fig. 5:**
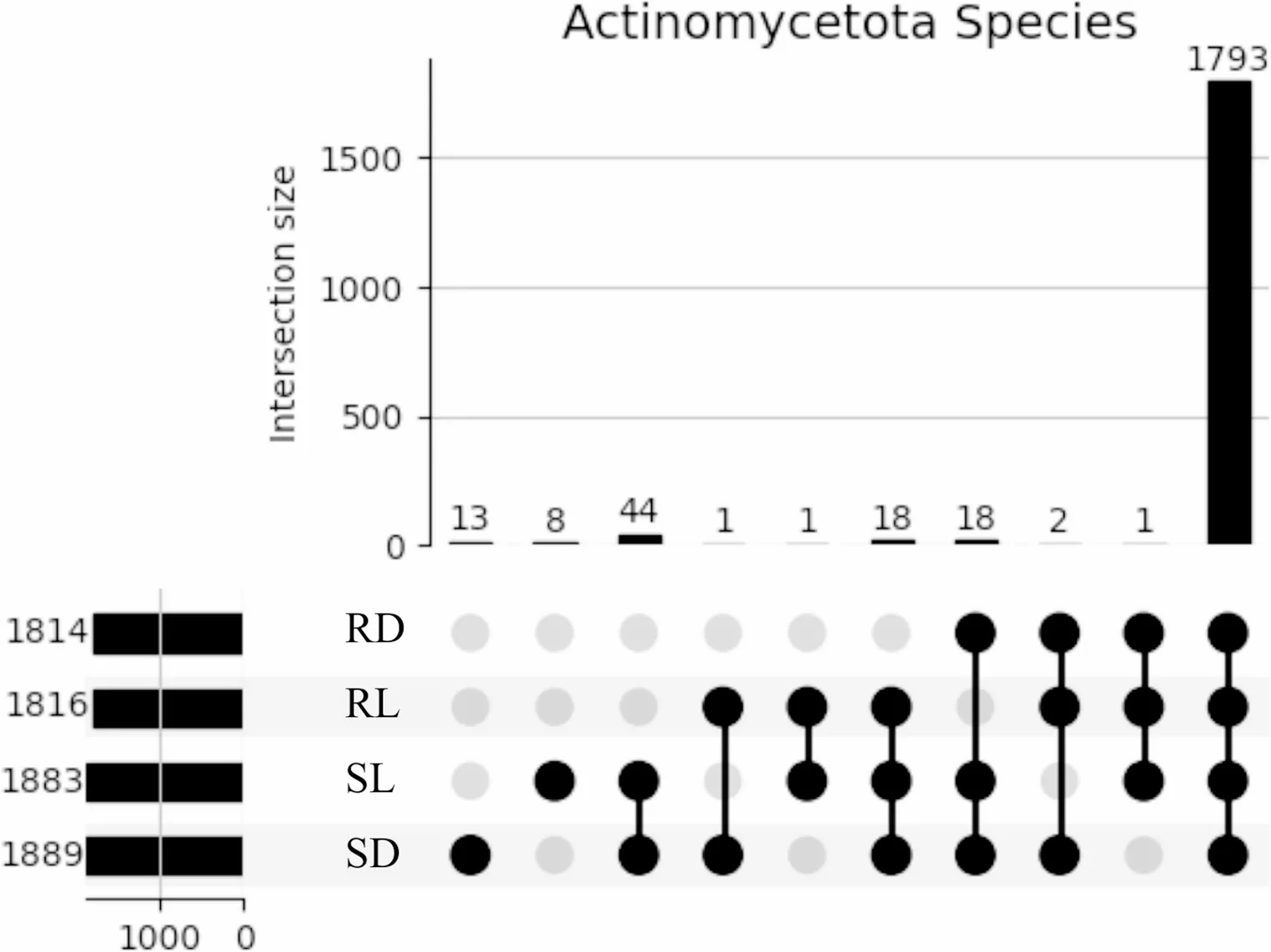
UpSet plot showing the overlap and exclusivity of *Actinobacteria* species among the analyzed samples. Bars represent the size of each intersection, while the matrix below indicates the presence (filled circles) or absence (empty circles) of species in each group

It was observed that, among the 1,899 *Actinobacteria* identified in this analysis (Fig. 5), 1,793 (n%) were common to all four sample groups (SD, SL, RL, and RD). In soil, 44 species were shared among bulk soil samples but were absent from the rhizosphere. In addition, 13 bacteria were exclusive to SD, and 8 were exclusive to SL. At the support-system level, only the rhizospheric soil with living support harbored the exclusive bacterium *Streptomyces* sp. 44,414.

On the other hand, there were 18 bacteria present in all samples but absent in RL, and another 18 absent only in RD.

The analysis of the group recruited from the soil by RL (Supplementary Material – Table III), but not by the rhizosphere with dead support, made it possible to observe the bacterial profile exclusive to this support system.

### Antimicrobial resistance genes

#### Diversity analyses

In the evaluation of the alpha diversity of ARGs using Fisher’s alpha and richness indices (Table 2), RL showed, descriptively, lower diversity and richness of resistance genes compared with the other groups. Overall, soil samples showed higher resistance gene richness than rhizosphere samples, suggesting a greater diversity of the resistance gene repertoire in soil.

**Table 2:** Mean of fisher’s alpha-diversity indices for resistance genes

Sample	Fisher	Richness
RD	0.89 ± 0.40	5.0
SD	0.93 ± 0.02	7.3
RL	0.36 ± 0.06	2.6
SL	0.91 ± 0.10	6.3

The alpha diversity of the identified resistance genes was higher in soil samples than in rhizosphere samples, as observed from the Fisher’s alpha values for SL (0.91 ± 0.10) and RL (0.36 ± 0.06). Among samples associated with dead support, the values were similar between SD (0.93 ± 0.02) and RD (0.89 ± 0.40). However, the pairwise comparisons performed using the Mann–Whitney U test did not identify statistically significant differences between any of the evaluated pairs (RD vs. RL, *p* = 0.1; RD vs. SD, *p* = 0.7; RD vs. SL, *p* = 1.0; RL vs. SD, *p* = 0.10; RL vs. SL, *p* = 0.10; SD vs. SL, *p* = 1.0). It is important to note that, with three replicates per group, the minimum p-value attainable by this method is *p* = 0.1. The Kruskal–Wallis test considering all four groups also detected no statistically significant differences in alpha diversity (H = 6.38; *p* = 0.094). Thus, although descriptive differences in diversity values were observed among the groups, these differences were not statistically confirmed.

#### Distribution of resistance genes

**Fig. 6:**
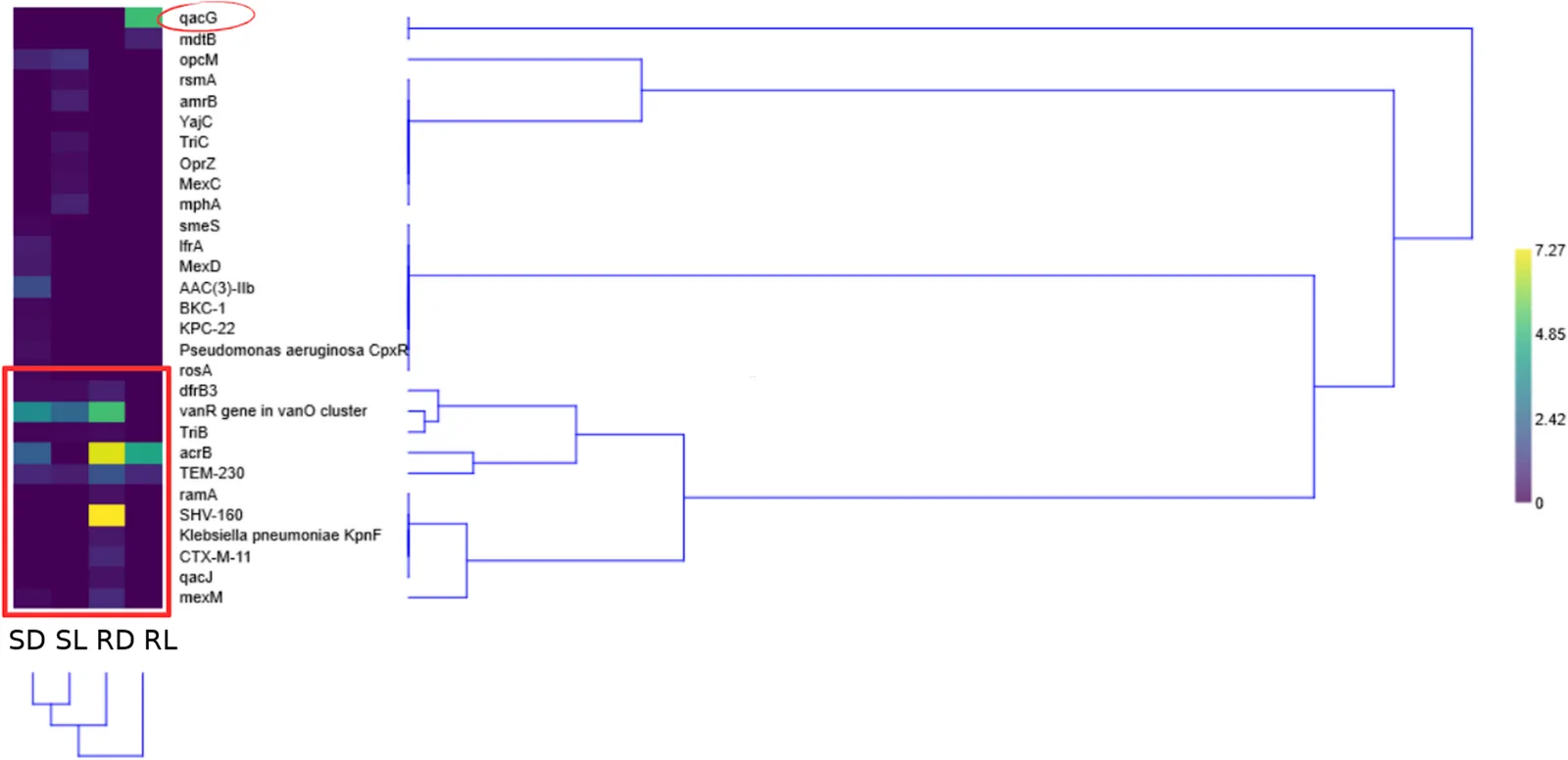
Similarity analysis of samples performed in orange data mining v. 3.38.1 based on the morisita distance matrix, highlighting the distribution of the qacG gene. clustering reveals patterns of similarity among the analyzed groups

The *acrB* gene, associated with an efflux pump that confers multidrug resistance, stands out in both rhizosphere samples (Fig. 6).

In RD, the genes *SHV-160* and *VanR* stand out due to their abundance and are associated with resistance to β-lactams and glycopeptides through antibiotic inactivation and target alteration, respectively.

On the other hand, RL shows a lower diversity of resistance genes, among which the *qacG* gene stands out due to its association with an efflux pump related to resistance to disinfectants and antiseptics.

In soil with a dead support (SD), there was a prevalence of genes related to resistance to aminoglycosides, glycopeptides, and multidrug resistance through the genes *AAC(3)-IIb*, *VanR*, and *acrB*. Similarly, in SL, the relative abundances of the *opcM* gene, an efflux pump of the RND family, and the *VanR* gene from the *VanO* cluster stand out.

#### Resistance mechanisms

**Fig. 7:**
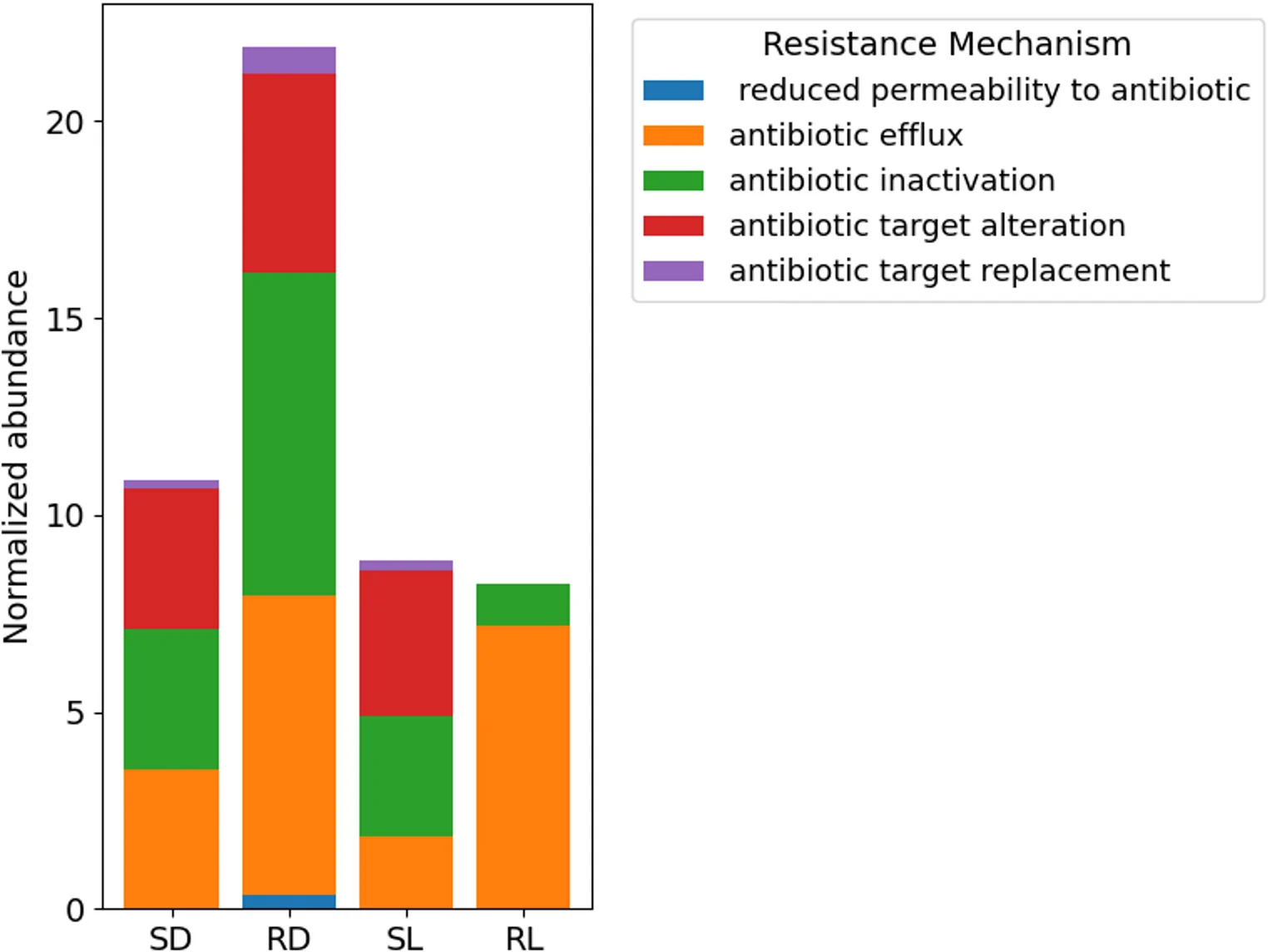
Stacked bar plot showing the average normalized distribution of resistance mechanism types across samples (RD, SD, SL, and RL). Colors represent different resistance mechanisms

Among the resistance mechanisms identified (Supplementary Material – Table V), a predominance of efflux pump-related mechanisms was observed in the rhizosphere of RL, whereas antibiotic inactivation followed by efflux pumps prevailed in RD (Fig. 7). In soil samples, mechanisms related to efflux, antibiotic inactivation, and target modification were the most prominent.

### AMRs profiles

**Fig. 8:**
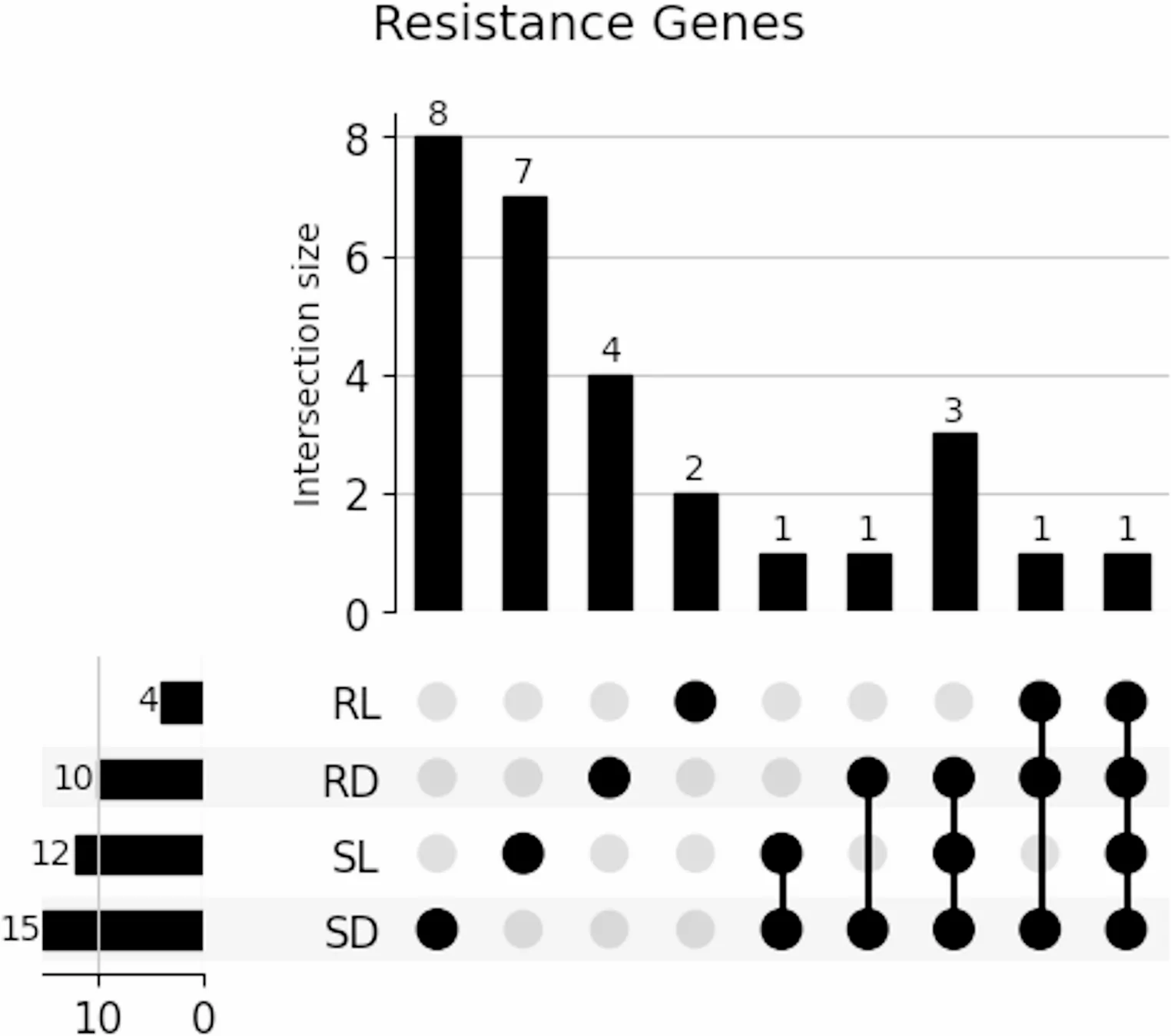
Analysis of intersections and exclusivities of resistance genes among the samples. Genes with a coverage of more than 75% were used to ensure coverage of at least two aligned reads per group

A total of 28 resistance genes were identified among the sample groups (Fig. 8). Based on the gene presence analysis (Supplementary Material – Table VI), no resistance genes exclusive to the live-support condition were observed, and only a single gene was found to be exclusive to the dead-support condition.

All samples shared the presence of *TEM-230*, a gene associated with resistance to β-lactam antibiotics, including monobactams, cephalosporins, penams, and penems, through antibiotic inactivation.

The *opcM* gene is associated with an efflux pump that confers resistance to fluoroquinolones and aminoglycosides. Regardless of the support system, the *opcM* gene was identified in bulk soil samples, whereas it was not detected in the rhizosphere groups.

The *dfrB3* gene was absent only in the RL sample (Table 3). This gene is associated with resistance to diaminopyrimidines through modification of the antibiotic target.

**Table 3:** Genes identified in bulk soil (SD and SL) and rhizospheric soil with dead support (RD), but not) butted in rhizospheric soil samples with living support (RL)

Gene	Drug Class	RD	RL	SD	SL
*TriB*	Disinfecting agents and antiseptics	Present	No detect	Present	Present
*dfrB3*	Disinfecting agents and antiseptics	Present	No detect	Present	Present
*vanR* gene in *vanO* cluster	Glycopeptide antibiotic	Present	No detect	Present	Present

It was also observed that the *VanR* gene of the *VanO* cluster, which is part of the cluster encoding resistance to vancomycin and teicoplanin, antibiotics commonly associated with *actinobacteria*, was absent only in the rhizosphere using the living support.

Regarding soil-exclusive genes, in SD, eight resistance genes were identified, with efflux and antibiotic inactivation mechanisms related to the RND, beta-lactamase, aminoglycoside, and MFS families. Among the seven genes observed in SL, there was a predominance of RND efflux pumps, with the exception of *mphA*, which confers resistance to macrolides through antibiotic inactivation.

In RD, a resistance profile composed of multidrug efflux pumps and a predominance of genes related to resistance to beta-lactams and disinfecting agents was observed. In RL, on the other hand, a reduced gene profile was noted, with only genes related to disinfecting agents and aminocoumarins (antibiotics derived from Streptomyces), suggesting possible resistance naturally induced by the presence of the producing bacterium.

### Association between variables

**Fig. 9:**
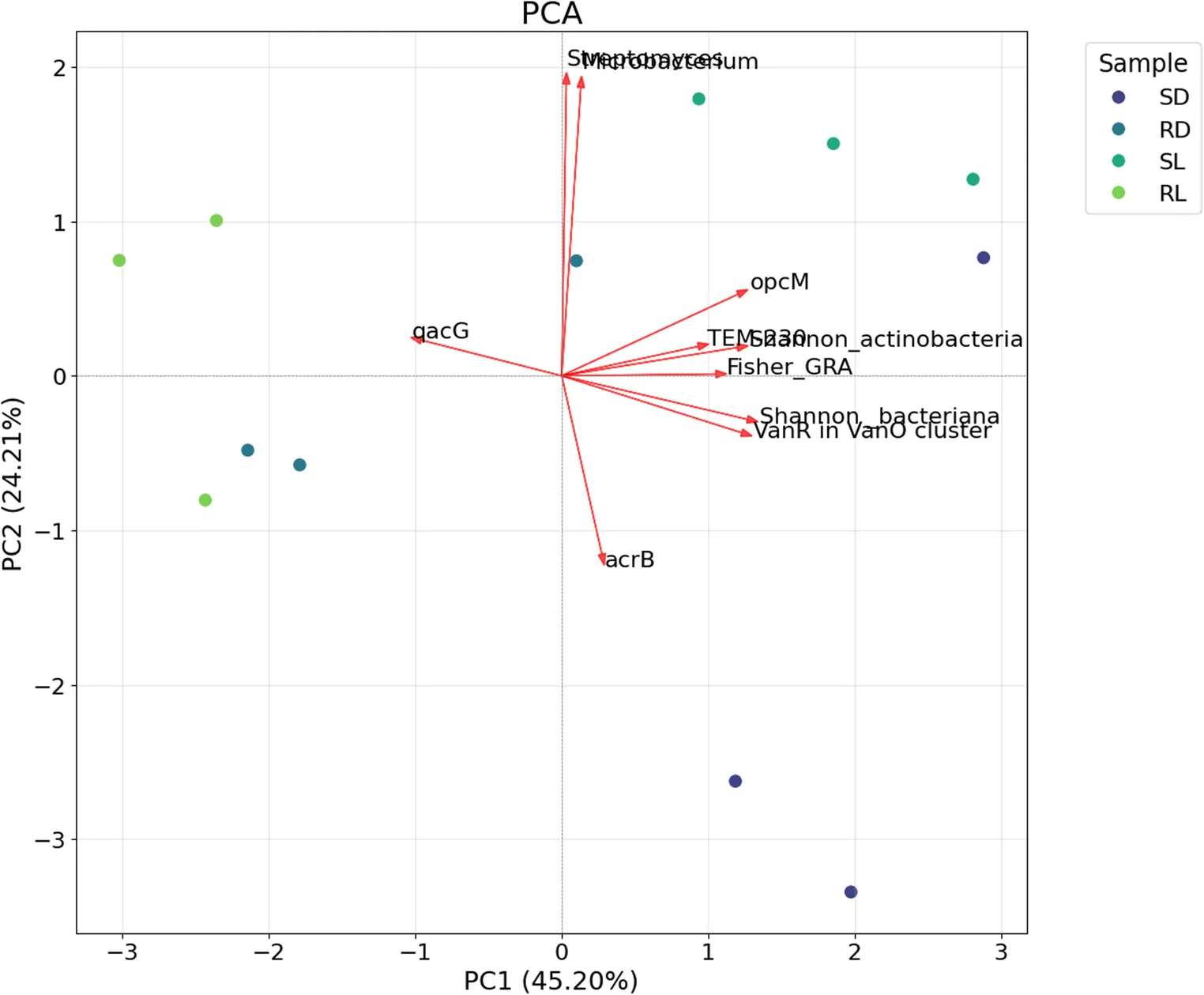
Principal Component Analysis (PCA) based on resistance genes, diversity indexes, and taxonomic composition across samples. The first two principal components explain 45.20% (PC1) and 24.21% (PC2) of the total variance. Colored points represent the different sample groups (SD, RD, SL, and RL). Red arrows indicate the variables contributing to sample separation, including resistance genes, diversity metrics, and dominant genera. The direction and length of each vector reflect the strength and orientation of its contribution to the ordination

Exploratory analysis (Fig. 9) of the variables in this study indicates that the occurrence and diversity of resistance genes are directly related to the structure and diversity of the bacterial community, which is reflected in the different resistance profiles observed. A strong correlation was observed between Fisher’s diversity of resistance genes and bacterial diversity (*R* = 0.60), as well as with the *VanR* gene (*R*= -.76), and a negative correlation with the resistance gene*qacG* (*R* = − 0.76), possibly because this gene shows higher prevalence in less competitive and functionally simpler environments.

Overall, the genes *VanR*, *TEM-230*, and *OpcM* are positively associated with bacterial diversity, actinobacterial diversity, and the Fisher diversity index of resistance genes.

Despite the high abundance of *Streptomyces* and its well-documented association with antibiotic production in the literature, no significant correlations were observed with the diversity of antimicrobial resistance (AMR) genes.

**Fig. 10:**
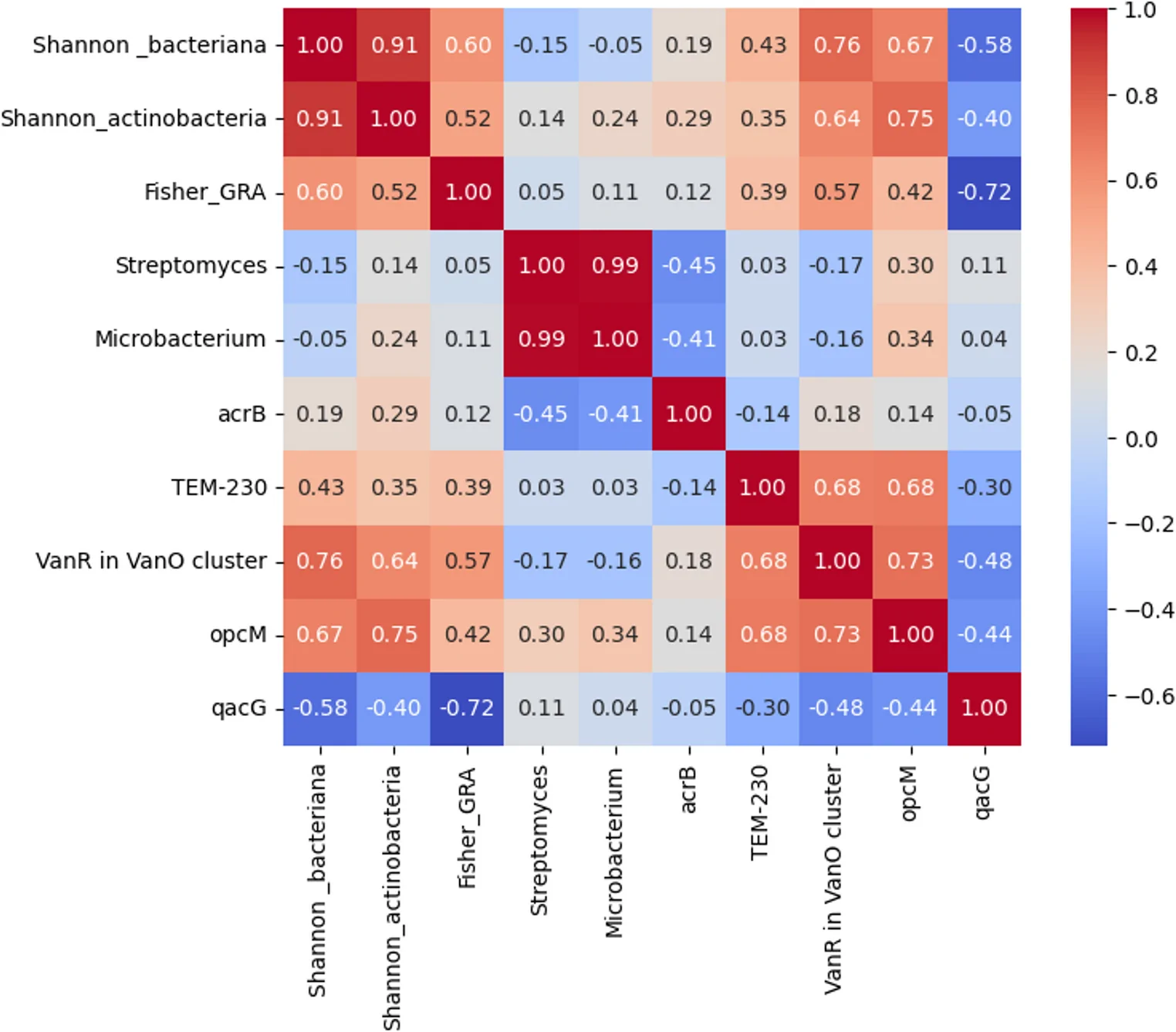
Pearson correlation matrix based on normalized data (StandardScaler). The heatmap shows correlations between bacterial diversity indices, Fisher’s alpha diversity of ARGs, selected bacterial taxa, and resistance genes. Color gradients indicate the strength and direction of correlations, ranging from strong negative (blue) to strong positive (red), with numerical values representing Pearson’s correlation coefficients (R)

The strong correlation observed between bacterial diversity and resistance gene diversity (Fig. 10) may indicate that, beyond antibiotic-producing bacteria belonging to the phylum Actinobacteria, additional mechanisms contribute to the dissemination of ARGs, such as horizontal gene transfer mediated by plasmids among bacterial populations.

### Molecular alterations

Gas chromatography-mass spectrometry (GC-MS) analysis resulted in the identification of eighty-one compounds (Table 4), corresponding to 98.26% and 99.24% of the total volatile chemical composition of fruits grown on dead and living support, respectively. The samples showed a predominance of monoterpenes hydrocarbons in both the dead (71.91 ± 9.61%) and living (75.46 ± 5.95%) support, followed by sesquiterpenes hydrocarbons (15.25 ± 5.51%) for dead support and (14.88 ± 3.22%) for living support.

**Table 4:** Volatile chemical composition of black pepper fruits cv. Bragantina, grown on living support (FTL) and dead support (FTD)

RI (C)	RI (L)	Compound	FTD	FTL
833	842	2-Methylpentanol	0.35 ± 0.50	0.19 ± 0.13
857	854	(2*E*) -Hexenol	1.34 ± 1.18	1.10 ± 0.94
905	914	3-Methylbutenol acetate	0.73 ± 1.03	0.00 ± 0.00
907	900	*n*-Nonane	0.17 ± 0.24	0.39 ± 0.35
923	920	(2*E*,4*E*) -Hexadienol	0.67 ± 0.94	0.00 ± 0.00
927	924	α-Thujene	1.12 ± 0.59	0.72 ± 0.62
936	932	α-Pinene	**15.55 ± 4.51**	**18.23 ± 0.66**
963	969	(*E*)-Pinano	0.21 ± 0.17	0.04 ± 0.06
979	974	β-Pinene	**28.55 ± 1.04**	**25.55 ± 1.45**
989	988	Myrcene	4.22 ± 3.20	7.31 ± 0.75
1008	1008	δ−3-Carene	0.23 ± 0.32	0.36 ± 0.51
1018	1018	α-Terpinene	0.00 ± 0.00	0.25 ± 0.18
1034	1024	Limonene	**21.46 ± 3.17**	**21.04 ± 1.45**
1047	1044	(*E*)-β-Ocimene	0.13 ± 0.08	0.20 ± 0.05
1059	1054	Ƴ-Terpinene	0.36 ± 0.03	0.55 ± 0.11
1068	1065	(*Z*)-Sabinene Hydrate	0.98 ± 0.27	0.62 ± 0.13
1079	1081	(2Z)-Hexenal. dietil acetal	0.21 ± 0.30	0.01 ± 0.01
1089	1098	(*E*)-Sabinene Hydrate	0.25 ± 0.36	0.00 ± 0.00
1090	1086	Terpinolene	0.47 ± 0.00	0.66 ± 0.08
1093	1100	*n*-Undecane	0.24 ± 0.18	0.00 ± 0.00
1094	1095	Linalool	1.48 ± 0.41	1.73 ± 0.40
1113	1111	4-Methyl-5-nonane	0.26 ± 0.37	0.00 ± 0.00
1115	1119	(*E*)-Pinene hydrate	0.13 ± 0.18	0.11 ± 0.08
1123	1124	(*Z*)-Menth-2-en-1-ol	0.00 ± 0.00	0.11 ± 0.03
1134	1132	(*Z*)-Limonene oxide	0.20 ± 0.14	0.05 ± 0.03
1140	1134	iso-3-Thujanol	0.11 ± 0.16	0.08 ± 0.03
1178	1174	4-Terpineol	1.40 ± 0.51	1.83 ± 0.49
1191	1186	α-Terpineol	0.37 ± 0.03	0.68 ± 0.11
1339	1335	δ-Elemene	0.12 ± 0.05	0.11 ± 0.03
1351	1345	α-Cubebene	0.28 ± 0.15	0.26 ± 0.07
1378	1374	α-Copaene	2.04 ± 0.67	1.96 ± 0.35
1392	1387	β-Cubebene	0.38 ± 0.19	0.35 ± 0.06
1394	1389	β-Elemene	0.37 ± 0.25	0.26 ± 0.13
1412	1409	α-Gurjunene	0.20 ± 0.11	0.20 ± 0.10
1417	1417	(*E*)-Caryophyllene	0.47 ± 0.19	5.78 ± 0.64
1457	1452	α-Humulene	0.76 ± 0.38	0.72 ± 0.22
1480	1484	Germacrene D	0.14 ± 0.13	0.12 ± 0.04
1489	1489	β-Selinene	0.57 ± 0.39	0.53 ± 0.23
1498	1496	Viridiflorene	0.61 ± 0.11	0.95 ± 0.35
1502	1500	α-Muurolene	0.21 ± 0.12	0.23 ± 0.07
1510	1505	β-Bisabolene	1.28 ± 0.61	1.46 ± 0.35
1519	1513	Ƴ-Cadinene	0.73 ± 0.51	0.69 ± 0.24
1526	1522	α-Cadineno	0.67 ± 0.38	0.78 ± 0.22
1587	1582	Caryophyllene oxide	0.43 ± 0.44	0.30 ± 0.18
1625	1627	*epi*-Cubenol	0.14 ± 0.11	0.14 ± 0.07
1641	1645	Cubenol	0.38 ± 0.29	0.05 ± 0.05
1646	1640	*epi*-α-Murrolol	0.17 ± 0.23	0.20 ± 0.16
1650	1651	α-Muurolol	0.88 ± 0.91	1.11 ± 0.44
Monoterpene hydrocarbons			71.91 ± 9.61	75.46 ± 5.95
Oxygenated monoterpenes			5.39 ± 1.82	5.35 ± 1.32
Sesquiterpene hydrocarbons			15.25 ± 5.51	14.88 ± 3.22
Oxygenated sesquiterpenes			2.22 ± 1.95	2.25 ± 1.05
Others			3.49 ± 4.10	1.30 ± 1.06
Total			98.26 ± 1.27	99.24 ± 0.81

*Compounds with concentrations below 0.10% were omitted from the table. RI(C) = “Retention Index” calculated in reference to a homologous series of n-alkanes. RI(L)= Literature Retention index

The fruits grown on both supports showed similar chemical profiles, with no significant quantitative differences. The main components identified in black pepper fruits, both in the dead and living support, were α-Pinene (15.55 ± 4.51 and 18.23 ± 0.66), β-Pinene (28.55 ± 1.04 and 25.55 ± 1.45), and Limonene (21.46 ± 3.17 and 21.04 ± 1.45).

**Fig. 11:**
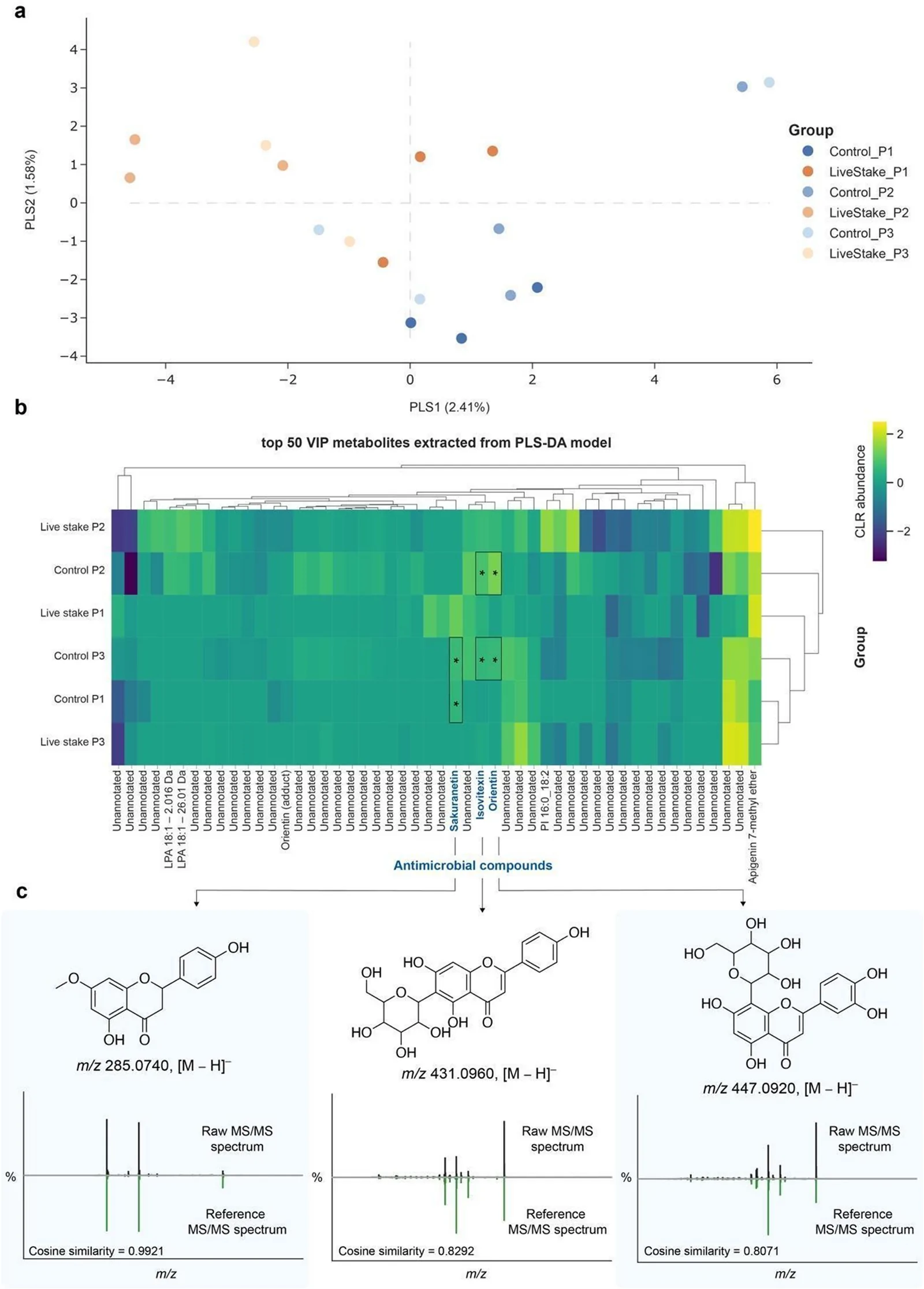
Metabolomic differentiation of *Piper nigrum* cultivated with dead and living support. (**a**) PLS-DA score plot showing clear separation between plants cultivated on dead support (Control) and living *Gliricidia sepium* support (living support), indicating support-dependent shifts in the plant metabolome. (**b**) Heatmap of the top 50 VIP metabolites from the PLS-DA model (centered log-ratio (CLR) abundance), highlighting three annotated flavonoid-derived compounds (sakuranetin, isovitexin, and orientin) enriched in dead-support plants. (**c**) Structural representations of these metabolites, along with mirror MS/MS matches against the GNPS reference library

The molecular analysis of *P. nigrum* fruits extracts revealed pronounced biochemical differences between pepper cultivated with dead support and those grown with living *Gliricidia sepium* (support). Although the support system did not strongly modify microbial diversity, the plant metabolome responded markedly to support type, producing distinct chemical fingerprints. The PLS-DA (Fig. 11a) clearly separated the two cultivation systems, indicating that support-related environmental conditions – nutrient cycling, shade modulation, soil microclimate, and microbial interactions – shape the secondary metabolism of *Piper nigrum*. Three metabolites were putatively annotated as sakuranetin (*m/z* 285.0740; spectral similarity = 99.21%) (Liu et al. 2023), isovitexin (*m/z* 431.0960; spectral similarity = 82.92%) (McNally et al. 2003), and orientin (*m/z* 447.0920; spectral similarity = 80.71%), corresponding to MSI level 2 annotations. These compounds belong to the flavonoid class and have been associated with antimicrobial and plant defense-related activities (Thongphichai et al. 2023). All three metabolites were strongly enriched in plants grown with dead support, whereas they were absent or detected at low abundance in plants grown with living support (Zhong et al. 2022).

The support type did not significantly affect the total phenolic content between plants cultivated with dead support and those grown with living support. However, it is noteworthy that the mean value of total phenolic content in RD is higher compared with RL, respectively 48.78 mg GAE.g-1 and 35.49 mg GAE.g-1.

## Discussion

### Bacterial diversity and composition

#### Bacterial diversity

The rhizosphere of plants has the ability to attract microorganisms of interest from the soil through the release of substances via their roots (rhizodeposits); these recruited microorganisms play important roles related to plant protection and nutrition (York et al. 2016; Knights et al. 2021). Although no significant differences in diversity resulting from the influence of the support system were detected, a finding corroborated by other studies that obtained similar results when evaluating microbial community differences in the rhizosphere of different plant species, this result should be interpreted with caution; the set of replicates used in the statistical analysis does not allow for the rejection of the null hypothesis (French et al. 2017; Fu et al. 2022). The study by Li and colleagues, conducted in four *P. nigrum* crops of different ages but with the same agronomic management, also did not show a statistical difference in alpha diversity, although it suggested differences in composition (Li et al. 2016).

The phylum *Actinomycetota* also did not show significant differences in diversity between support systems. The homogeneity of the bacterial diversity results, especially for the phylum *Actinobacteria*, can be attributed to the geographic distance between the sampling points in this study and the strong dispersal capacity and resilience of bacterial taxa (Araújo et al. 2020; Na et al. 2018).

Based on this, the choice of support system did not show evidence of being a risk factor for the microbial diversity evaluated in this study. We hypothesize that the replacement of the dead support, a more costly and less sustainable method (EMBRAPA, 2004), with *G. sepium* support did not cause negative impacts on microbial alpha-diversity indices, including the phylum *Actinobacteria*.

#### Bacterial composition

In addition, there is evidence that several species within these phyla are associated with the synthesis of molecules capable of promoting plant growth, such as auxins, as well as the production of antibiotics that help protect against plant pathogens such as *Fusarium*, *P. capsici*, and *S. rolfsii* (Wang 2023; Anusree and Suseela Bhai 2017).

The prevalence of the family *Streptomycetaceae* in RD may indicate that these bacteria are playing roles in plant development. The lower abundance of *Pseudomonadaceae* in RL may indicate that the presence of the *G. sepium* support, known for its contribution to soil fertility, influences the recruitment of members of this family (Florentino et al. 2014).

Furthermore, the removal of the most abundant families as extreme data allowed greater differences in the compositional profiles of the groups to be observed, suggesting that bacteria performing functions specific to the different groups may not be among the most abundant. The genus *Streptomyces* is frequently identified in soil samples (Karthik et al. 2022), which may be related to the biological functions performed by different *Streptomyces* species in this environment, such as the production of natural antibiotics, antimicrobial peptides, siderophores, phytohormones, and others (Anusree et al. 2019; Gowdar et al. 2018). Thus, the recruitment of this genus may be associated with protection against phytopathogens, as well as with increased nutrient availability and the promotion of plant growth.

### Profiles of actinobacteria species

Interestingly, Thapa et al. (2024) performed a molecular comparison among different *Streptomyces* strains, and their results indicated that, regardless of geographic variation, there may be a high similarity among the metabolites produced by bacteria of this genus when grown under the same conditions (Thapa et al. 2024). This could indicate that, although rhizospheres recruit different *Streptomyces* assemblages, these bacteria may perform similar functions in the environment. A more in-depth analysis of the metabolism of these bacteria across the different samples would be necessary to determine how much influence the rhizosphere and the support system have on inducing metabolite production by *Streptomyces*.

Among the group recruited by RL, species associated with the biocontrol of phytopathogens, the production of bioactive compounds, the degradation of pollutants such as heavy metals, and the synthesis of compounds of agricultural importance were observed (Wang et al. 2021). The literature highlights *Streptomyces griseorubiginosus*,* Rhodococcus sp. 11 − 3*,* Paenarthrobacter sp. R1*, and *Microbacterium sp. AIS03* for their production of plant growth-promoting secondary metabolites (Chai et al. 2022), biocontrol of pathogens, increased nutrient availability in soil, and bioremediation of pollutants such as organophosphate compounds and heavy metals (Xiao et al. 2022; Iyer et al. 2017).

Thus, it is possible that the RL condition contributes to the recruitment of bacteria associated with the production of plant growth-promoting compounds and mechanisms of protection against phytopathogens, such as the regulation of the plant’s enzymatic defense response. The presence of *G. sepium* could also favor bacteria that aid plant development, corroborating the previous study by Dinesh et al. (2010), which reported increased microbial biomass and nutrient availability in soil with *G. sepium* support.

The Actinobacteria recruited in RD (Supplementary Material – Table IV) are characterized by the biosynthesis of and resistance to a range of antibiotics, including carbapenems, monobactams, chloramphenicol, and streptomycin (Caradec et al. 2022; Chen et al. 2020). Among the bacteria recruited in RD are *Dactylasporangium fulvum*, *Streptomyces sp. S501*, and *Nocardioides sp. LMS-CY* (Cheng et al. 2021), which may be involved in the dissemination of ARGs. The species *Streptomyces sp. 44,414*, identified in RL samples, has not been reported to be directly associated with the biosynthesis of antimicrobial compounds. However, this bacterium may exhibit functions related to gene transfer among species through the intramycelial dissemination of plasmids (Zhang et al. 2008).

### Antimicrobial resistance genes

#### Diversity analyses

This pattern may suggest that the rhizosphere community harbors a more restricted repertoire of resistance genes, potentially associated with the selection and recruitment of specific microbial groups within the rhizosphere environment.

These results suggest a possible role for *G. sepium* in the selection of resistance genes recruited to the rhizosphere under living support treatment conditions. However, further studies with a larger number of replicates are needed to better understand how this plant contributes to black pepper development, particularly with regard to microbial recruitment, and to increase the statistical power of the analyses.

#### Distribution of resistance genes

The abundance patterns of antimicrobial resistance genes (Fig. 6) suggest that the microbial structure in the different groups plays a fundamental role in the selection of resistance genes. The *acrB* gene is widely identified in different environments, including minimally impacted soils, such as Antarctic soil (Yuan et al. 2019). Its high prevalence may be related to the fact that this gene confers resistance to a broad range of antibiotics, providing bacteria with a versatile defense mechanism.

In addition, Nishino et al. (2021) indicate that *acrB* plays a role in the excretion of the siderophore enterobactin to the exterior of the bacterium; this siderophore is released as a mechanism to facilitate iron acquisition from the environment (Nishino et al. 2021). The secretion of siderophores has been highlighted in studies as one of the mechanisms of interest in plant growth–promoting bacteria due to their ability to increase iron bioavailability for plants through the chelation of inorganic Fe³⁺ (Sun et al. 2022). Although the involvement of this gene has been reported in pathogenic *E. coli* (Nishino et al. 2021), it is possible that it may perform different biological functions in environmental bacteria present in agricultural systems.

The distribution of resistance genes observed in RD suggests that this group possesses greater resistance diversity, possibly associated with increased recruitment of species potentially related to the production of antibiotics that may act as inhibitors of phytopathogens (Yasir et al. 2022; Alonso-Reys et al. 2021).

The higher abundance of the *qacG* gene in the rhizosphere with a living support may indicate a greater demand for this gene in the adaptation of microbial communities associated with this cultivation system, possibly as a result of the application of biocides or the disinfection protocol for *G. sepium* seedlings.

Based on gene abundance and richness, the RL sample presents a resistance profile distinct from the other samples, which suggests the influence of the living support on the selection of ARGs in the rhizosphere. In addition, through gene clusters, it is possible to associate this difference not only with the *qacG* gene, whose incidence occurs only in RL, but also with the greater variety and abundance of ARGs in the other samples.

Previous analyses of the presence of *Actinomycetota* may indicate that the rhizosphere with a dead support harbors a greater number of exclusive bacteria related to antibiotic production and resistance compared with the living support, whose exclusive bacteria are often associated with plant growth promotion (Chai et al. 2022; Wang et al. 2021). The higher diversity of resistance genes observed in the RD sample may be associated with differences in the recruitment of actinobacteria by rhizospheres with living versus dead supports.

Therefore, we hypothesize that the presence of the living support *Gliricidia sepium* may be influencing the selection of ARGs by partially meeting the metabolic demands of the microbiome, which could reduce the selective pressure for increases in the abundance of these genes (Juanico et al. 2023). In contrast, in the absence of the living support, unmet metabolic demands may increase selective pressure, potentially leading to higher abundances of these genes and favoring their persistence and dissemination in the environment.

Thus, resistance genes in agricultural soils should not be interpreted solely from the perspective of antimicrobial resistance related to anthropogenic impact, but their participation in the fundamental metabolism of soil microbial communities and the interactions among them should also be considered. Accordingly, these genes may play a role in nutrient availability and microbial competition. Based on these results, it is suggested that the resistance profile in RD may be driven by greater niche competition due to lower nutrient availability compared with the living support system, which includes *G. sepium* in addition to black pepper for the secretion of substances into the rhizospheric horizon.

#### Resistance mechanisms

Efflux pumps are an important mechanism for bacterial survival in environments under selective pressure and are therefore widely disseminated across diverse environments. Thus, considering that these genes have an evolutionary history that predates the anthropogenic use of antimicrobials, as suggested by studies conducted in Antarctic soils (Yuan et al. 2019), the presence of efflux pump-related genes is not necessarily an indicator of antibiotic contamination caused by human activity, but rather of natural resistance induced by the presence of antibiotic-producing microorganisms (Ramakrishna et al. 2019).

In addition to bacterial survival against antimicrobials, efflux pumps are involved in several bacterial metabolic functions, such as the excretion of substances that solubilize nutrients like iron (Nishino et al. 2021). Their higher incidence in rhizospheric samples compared to bulk soil samples may indicate that this mechanism plays roles related to nutrient availability captured by plant roots.

#### AMRs profiles

The *TEM-230* gene belongs to the *TEM* beta-lactamase family and is commonly identified in enterobacteria belonging to the phylum *Pseudomonadota* (Ranjbar and Sami 2017).

The absence of the *opcM* gene in the rhizosphere may indicate that microorganisms carrying this gene are not involved in rhizospheric metabolism or plant growth promotion.

Although the *dfrB3* gene has been reported at higher proportions in soils without cattle manure fertilization (Macedo et al. 2021), all samples evaluated in this study were managed using castor cake fertilizer. The study by Kneis et al. (2023) indicates the possibility that some copies of this gene are part of resistance cassettes; thus, this gene may be present in the sample not due to activation by the diaminopyrimidine stressor, but rather due to the co-selection mechanism of other genes (Kneis et al. 2023).

The absence of the *VanR* gene in RL may be related to the fact that the group of actinobacteria exclusively recruited by RL is mainly composed of bacteria associated with plant growth promotion (Binda et al. 2018).

This pattern strengthens the hypothesis that RL is less conducive to the maintenance of ARGs, favoring plant growth-promoting microorganisms over those involved in chemical antagonism. Similarly, the more diverse genetic framework in RD suggests a more competitive environment, which may require a larger microbial defense arsenal.

Other mechanisms, beyond selection pressure due to the presence of antibiotic-producing bacteria, may be involved in the persistence of resistance genes in the environment, such as cross-resistance, co-resistance, and co-regulation (Maurya et al. 2020). Biocides (frequently used in crops) and disinfectants, for example, can also select resistant strains as a consequence of the expression of multidrug resistance genes, and even genes directly associated with antibiotics may be selected if they are part of the same replicon carrying other genes of interest (Alonso et al. 2001; Cárdenas-Alegría et al. 2022a, 2022b).

Overall, the observed presence-absence patterns of resistance genes in the samples indicate that the support system may influence the functional structure of the black pepper rhizosphere. The lower diversity of these genes in RL suggests that this environment may be chemically less competitive, potentially favoring the recruitment of plant growth-promoting microorganisms, with the actinobacterial community recruited in this sample standing out as being predominantly associated in the literature with plant-beneficial functions (Chai et al. 2022; Xiao et al. 2022; Iyer et al. 2017).

#### Association between variables

The presence of the support plant *G. sepium*, and the potential increase in exudate availability, may be mediating a reduction in competition among bacteria for ecological niches, favoring communities that are less dependent on antimicrobial resistance mechanisms.

The correlation between the *VanR*, *TEM-230*, and *OpcM* genes and actinobacteria may suggest that they are related to more complex and nutritionally competitive microbial communities, in which AMR genes may be performing multiple adaptive functions.

The absence of significant correlations between *Streptomyces* and resistance genes suggests that this genus may perform other ecological functions within this niche. Moreover, its presence does not necessarily imply increased AMR diversity. Nevertheless, it is important to highlight that these bacteria have the potential to synthesize a wide range of antibiotics. Overall, the results indicate that factors other than the presence of microorganisms traditionally known for antibiotic production, such as *Streptomyces*, may play a more prominent role in driving the diversity of resistance genes (Rad et al. 2022).

Additional mechanisms, such as plasmid-mediated horizontal gene transfer among bacterial populations, may contribute to the dissemination of ARGs. This gene transfer mechanism may pose a risk to agricultural cultivation systems under strong selective pressures, potentially leading to the emergence of opportunistic or pathogenic strains (Dimitriu 2022; Meng et al. 2022).

Similarly, the moderate correlation between Actinobacteria and the diversity of antimicrobial resistance genes supports previous reports indicating that antibiotic-producing organisms tend to harbor a broad genetic repertoire of AMRs as a strategy to ensure their own survival. Nevertheless, these results also suggest that additional factors may play a substantial role in shaping AMR diversity, including the contribution of other bacterial phyla, fungi, mobile genetic elements (MGEs), and bacteriophages (Stanczak-Mrozek et al. 2017; Fisher et al. 2022).

#### Molecular alterations

The chemical composition is in agreement with previous studies, which showed a content of 79.23% of monoterpenes hydrocarbons in *P. nigrum* cv. Bragantina fruits, with α-Pinene (10.67%), β-Pinene (35.05%), and Limonene (21.67%) being the major components (Barata et al. 2021).

The main compounds identified in this study are consistent with those described for most black pepper cultivars, as widely reported in the literature (Dosoky et al. 2019). Terpene levels and profiles exert a direct and significant influence on the aroma and flavor of the fruits, with these sensory attributes being closely associated with the presence and proportion of these compounds, regardless of the geographical region of cultivation (Zachariah et al. 2010).

Additionally, the results obtained in this work show high similarity to those reported for fruits marketed in Shanghai, China, in which β-caryophyllene was identified as the major component (23.49%), followed by 3-carene (22.20%), D-limonene (18.68%), β-pinene (8.92%), α-phellandrene (5.5%), α-pinene (4.4%), eugenol (4.0%) and β-myrcene (2.8%) (Liu et al. 2007).

Because flavonoids such as sakuranetin, isovitexin, and orientin are classical indicators of induced plant defense (Ramaroson et al. 2022), their accumulation suggests that dead-support systems impose greater physiological, microbial, or environmental stress on the plant (Ullah et al. 2025), thereby activating phenylpropanoid-mediated defense pathways (Sharma et al. 2019).

This biochemical pattern aligns tightly with the agronomic and ecological dynamics inferred from the microbiome and resistome profiles. Dead-support rhizospheres (RD) displayed a higher abundance of potential antibiotic-producing *Actinobacteria* and a richer repertoire of AMR genes, while pepper plants in the same system accumulated more antimicrobial secondary metabolites (Reimer et al. 2022). This convergence suggests a feedback loop in which plant stress elevates defensive metabolite production, which in turn selects for resistant and antagonistic microbial communities, reinforcing AMR enrichment. Conversely, the living-support system (RL) exhibited lower levels of plant defense metabolites, fewer AMRs, and a reduced presence of taxa known to include antibiotic-producing actinobacteria. These features indicate a more stable and cooperative soil-plant-microbe environment, likely supported by *Gliricidia*’s contributions to nutrient cycling, microclimate buffering, and recruitment of beneficial microorganisms.

The result of the total phenolic content analysis corroborates the analysis of the extracts’ chemical profile by LC-MS/MS, considering that phenolic compounds play a key role in plant defense systems against pathogens, and some substances, such as simple phenols and phenolic phytoalexins, are associated with stress response (SALUNKE and KOCHE, 2023).

Together, these results demonstrate that the choice of support system has consequences far beyond structural support: dead support create a more stressful ecological context that elicits chemical defenses in pepper plants and enriches resistance-associated microbial functions (Liu et al. 2023; McNally et al. 2003), whereas living support mitigate stress and suppress the need for costly secondary metabolism. The integration of molecular profile, microbiome, and AMR data provides a cohesive picture in which *Gliricidia* acts not only as an agronomically sustainable support but also as a stabilizing biological element that promotes plant health and reduces ecological pressure on the rhizosphere.

## Conclusion

This study revealed that Actinobacteria are present in high proportions across all samples, with greater diversity in bulk soil compared to the rhizosphere. Although the overall bacterial diversity was not strongly influenced by the type of support, the composition of the community and the selection of antimicrobial resistance genes (AMRs) showed clear support-dependent patterns. Dead-support rhizospheres (RD) harbored a broader repertoire of ARGs and a higher abundance of possible antibiotic-producing Actinobacteria, whereas living-support rhizospheres (RL) exhibited reduced ARGs diversity, suggesting that the presence of *Gliricidia sepium* may partially substitute ecological functions typically associated with these microbial groups.

Metabolomic results further demonstrated that the support system influences *Piper nigrum* physiology at the molecular level. Plants grown on dead support showed a pronounced accumulation of defense-related flavonoids - including sakuranetin, isovitexin, and orientin - compounds widely recognized for their antimicrobial and stress-responsive roles. The absence or low abundance of these metabolites in plants cultivated with living support indicates that *G. sepium* reduces physiological and environmental stress, likely through improved nutrient availability, microclimate buffering, and beneficial microbial recruitment. The convergence of metabolomic, microbiome, and resistome data suggests a feedback mechanism in which stress experienced in dead-support systems induces defensive secondary metabolism, which in turn selects for resistant or antagonistic microbial communities, reinforcing AMR enrichment.

Together, these findings suggest that the choice of support system has consequences that extend beyond mechanical plant support. Dead support appears to create a more stressful ecological context that stimulates plant defense pathways and favors the persistence of resistance-associated microbial functions, whereas living support promotes a more stable soil–plant–microbe environment and is associated with reduced resistome diversity. In addition to reducing deforestation and lowering input costs, the adoption of G. sepium as a living support may therefore enhance the health and productivity of black pepper cultivation by mitigating plant stress and fostering a more balanced rhizosphere. Further research is needed to determine how these microbial and chemical interactions ultimately influence long-term crop resilience and yield.

## Supplementary Information

Below is the link to the electronic supplementary material.

Supplementary File 1 (DOCX 15.4 KB)

Supplementary File 2 (DOCX 280 KB)

## Data Availability

The sequence data are available on NCBI under Bioproject PRJNA1295359.

## References

[CR2] Adams RP (2012) Identification of essential oil components by gas chromatography/mass spectrometry. Allured Business Media

[CR3] Alcock BP, Huynh W, Chalil R, Smith KW, Raphenya AR, Wlodarski MA, Edalatmand A, Petkau A, Syed SA, Tsang KK, Baker SJC, Dave M, McCarthy MC, Mukiri KM, Nasir JA, Golbon B, Imtiaz H, Jiang X, Kaur K, McArthur AG (2023) CARD 2023: expanded curation, support for machine learning, and resistome prediction at the Comprehensive Antibiotic Resistance Database. Nucleic Acids Res 51(D1):D690–D699. 10.1093/nar/gkac920 PMC9825576 36263822

[CR4] Alonso A, Sánchez P, Martínez JL (2001) Environmental selection of antibiotic resistance genes. Environ Microbiol 3(1):1–9. 10.1046/j.1462-2920.2001.00161.x 11225718

[CR5] Alonso-Reyes DG, Galván FS, Portero LR, Alvarado NN, Farías ME, Vazquez MP, Albarracín VH (2021) Genomic insights into an andean multiresistant soil actinobacterium of biotechnological interest. World J Microbiol Biotechnol 37(10):166. 10.1007/s11274-021-03129-9 PMC8405860 34463818

[CR6] Anusree T, Suseela Bhai R (2017) Rhizosphere actinobacteria for combating Phytophthora capsici and Sclerotium rolfsii, the major soil borne pathogens of black pepper (*Piper nigrum L*). Biol Control 109:1–13. 10.1016/j.biocontrol.2017.03.006

[CR7] Anusree T, Suseela Bhai R, Shabeer A, T. P., Oulkar D (2019) Streptomyces spp from black pepper rhizosphere: a boundless reservoir of antimicrobial and growth promoting metabolites. J Biologically Act Prod Nat 9(1):1–23. 10.1080/22311866.2018.1561327

[CR8] Araujo R, Gupta VVSR, Reith F, Bissett A, Mele P, Franco CMM (2020) Biogeography and emerging significance of Actinobacteria in Australia and Northern Antarctica soils. Soil Biol Biochem 146:107805. 10.1016/j.soilbio.2020.107805

[CR9] Araújo E, Gonçalves Júnior M, Guerra J, Espindola J, Alves B, Correia M, Risso I, Santos S, Silva C, Rezende A, Mello G, Santos C, Silva F (2024) Comunicado Técnico Sistema mecanizado de produção de biomassa de gliricídia

[CR10] Barata LM, Andrade EH, Ramos AR, de Lemos OF, Setzer WN, Byler KG, Maia JGS, da Silva JKR (2021) Secondary metabolic profile as a tool for distinction and characterization of cultivars of black pepper (Piper nigrum L.) Cultivated in Pará State, Brazil. Int J Mol Sci 22(2):890. 10.3390/ijms22020890 PMC7830865 33477389

[CR12] Binda E, Cappelletti P, Marinelli F, Marcone GL (2018) Specificity of induction of glycopeptide antibiotic resistance in the producing actinomycetes. Antibiotics 7(2):36. 10.3390/antibiotics7020036 PMC6022977 29693566

[CR13] Bolger AM, Lohse M, Usadel B (2014) Trimmomatic: a flexible trimmer for Illumina sequence data. Bioinf (Oxford England) 30(15):2114–2120. 10.1093/bioinformatics/btu170 PMC4103590 24695404

[CR15] Breitwieser FP, Salzberg SL (2020) Pavian: interactive analysis of metagenomics data for microbiome studies and pathogen identification. Bioinformatics 36(4):1303–1304. 10.1093/bioinformatics/btz715 PMC8215911 31553437

[CR16] Caradec T, Trivelli X, Desmecht E, Peucelle V, Khalife J, Hartkoorn RC (2022) Dactylosporolides: glycosylated macrolides from dactylosporangium fulvum. J Nat Prod 85(12):2714–2722. 10.1021/acs.jnatprod.2c00484 PMC9791991 36512509

[CR18] Cardenas Alegria O, Pires Quaresma M, Dias Dantas CW, Silva Guedes Lobato EM, de Oliveira Aragão A, Patroca da Silva S, Costa Barros da Silva A, Ribeiro Cruz AC, Ramos RTJ, Carneiro AR (2022a) Impacts of soybean agriculture on the resistome of the Amazonian soil. Front Microbiol 13. 10.3389/fmicb.2022.948188 PMC9500545 36160259

[CR19] Cardenas-Alegria OV, Dantas CWD, Silva Guedes Lobato EM, da Silva SP, Moraes C, de Cruz S, Ramos ACR, R. T. J., Carneiro AR (2022b) Influence of soil management for soybean production under microbial diversity in amazon soils. Front Environ Sci 10. 10.3389/fenvs.2022.1024610

[CR20] Chai CH, Hong C-F, Huang J-W (2022) Identification and characterization of a multifunctional biocontrol agent, *Streptomyces griseorubiginosus* LJS06, against cucumber anthracnose. Front Microbiol. 10.3389/fmicb.2022.923276 PMC9201727 35722317

[CR21] Chambers MC, Maclean B, Burke R, Amodei D, Ruderman DL, Neumann S, Gatto L, Fischer B, Pratt B, Egertson J, Hoff K, Kessner D, Tasman N, Shulman N, Frewen B, Baker TA, Brusniak M-Y, Paulse C, Creasy D, Flashner L, Kani K, Moulding C, Seymour SL, Nuwaysir LM, Lefebvre B, Kuhlmann F, Roark J, Rainer P, Detlev S, Hemenway T, Huhmer A, Langridge J, Connolly B, Chadick T, Holly K, Eckels J, Deutsch EW, Moritz RL, Katz JE, Agus DB, MacCoss M, Tabb DL, Mallick P (2012) A cross-platform toolkit for mass spectrometry and proteomics. Nat Biotechnol 30:918–920 10.1038/nbt.2377 PMC3471674 23051804

[CR22] Chen C, Chen S, Zhang W, Yuan F, Yu J, Liu Q (2020) Streptomyces sp. S501, a marine petroleum-degrading actinobacterium isolated from sediment of Yalujiang Estuary, Northern China, and Its Genome Annotation. Curr Microbiol 77(11):3643–3650. 10.1007/s00284-020-02181-2 32895802

[CR23] Cheng M, Qian Y, Xing Z, Zylstra GJ, Huang X (2021) The low-nanomolar 4-nitrobenzoate-responsive repressor PnbX negatively regulates the actinomycete-derived 4-nitrobenzoate-degrading pnb locus. Environ Microbiol 23(11):7028–7041. 10.1111/1462-2920.15787 34554625

[CR24] Chieb M, Gachomo EW (2023) The role of plant growth promoting rhizobacteria in plant drought stress responses. BMC Plant Biol 23(1):407. 10.1186/s12870-023-04403-8 PMC10464363 37626328

[CR26] Dimitriu T (2022) Evolution of horizontal transmission in antimicrobial resistance plasmids. Microbiology (Reading). 10.1099/mic.0.001214 35849537

[CR27] Dinesh R, Srinivasan V, Hamza S, Parthasarathy VA, Aipe KC (2010) Physico-chemical, biochemical and microbial properties of the rhizospheric soils of tree species used as supports for black pepper cultivation in the humid tropics. Geoderma 158(3–4):252–258. 10.1016/j.geoderma.2010.04.034

[CR29] Dosoky NS, Satyal P, Barata LM, da Silva JKR, Setzer WN (2019) Volatiles of black pepper fruits (*Piper nigrum L*). Molecules 24(23):4244. 10.3390/molecules24234244 PMC6930617 31766491

[CR32] EMBRAPA (2004) Manual segurança e qualidade para a cultura da pimenta-do-Reino

[CR33] Fiehn O, Robertson D, Griffin J, van der Werf M, Nikolau B, Morrison N, Sumner LW, Goodacre R, Hardy NW, Taylor C, Fostel J, Kristal B, Kaddurah-Daouk R, Mendes P, van Ommen B, Lindon JC, Sansone S-A (2007) The metabolomics standards initiative (MSI). Metabolomics 3(3):175–178. 10.1007/s11306-007-0070-6

[CR36] Fisher MC, Alastruey-Izquierdo A, Berman J, Bicanic T, Bignell EM, Bowyer P, Bromley M, Brüggemann R, Garber G, Cornely OA, Gurr SJ, Harrison TS, Kuijper E, Rhodes J, Sheppard DC, Warris A, White PL, Xu J, Zwaan B, Verweij PE (2022) Tackling the emerging threat of antifungal resistance to human health. Nat Rev Microbiol 20(9):557–571. 10.1038/s41579-022-00720-1 PMC8962932 35352028

[CR37] Florentino L, Rezende A, Mesquita A, Lima A, Marques D, Miranda J (2014) Diversity and potential use of rhizobia from nodules of *Gliricidia sepium.* Revista de Ciências Agrárias, 37 n3(2019-01-. 20320–328. 10.19084/rca.16831

[CR39] French KE, Tkacz A, Turnbull LA (2017) Conversion of grassland to arable decreases microbial diversity and alters community composition. Appl Soil Ecol 110:43–52. 10.1016/j.apsoil.2016.10.015

[CR41] Fu L, Yan Y, Li X, Liu Y, Lu X (2022) Rhizosphere soil microbial community and its response to different utilization patterns in the semi-arid alpine grassland of northern Tibet. Front Microbiol. 10.3389/fmicb.2022.931795 PMC9354816 35935214

[CR43] Gowdar SB, Deepa H, Amaresh YS (2018) A brief review on biocontrol potential and PGPR traits of Streptomyces sp. for the management of plant diseases. J Pharmacognosy Phytochemistry 7(5)

[CR107] IBGE (2023) Produção de Pimenta-do-reino. https://www.ibge.gov.br/explica/producao-agropecuaria/pimenta-do-reino/br

[CR49] Inda-Díaz JS, Lund D, Parras-Moltó M, Johnning A, Bengtsson-Palme J, Kristiansson E (2023) Latent antibiotic resistance genes are abundant, diverse, and mobile in human, animal, and environmental microbiomes. Microbiome 11(1):44. 10.1186/s40168-023-01479-0 PMC9993715 36882798

[CR50] Iyer R, Damania A, Iken B (2017) Whole genome sequencing of Microbacterium sp. AISO3 from polluted San Jacinto River sediment reveals high bacterial mobility, metabolic versatility and heavy metal resistance. Genomics Data 14:10–13. 10.1016/j.gdata.2017.07.009 PMC5542380 28794988

[CR51] Juanico MP, Purnamasari L, Hwang SG, dela Cruz JF (2023) The potential of *Gliricidia sepium* plant extract as antibacterial and antifungal: a review. Eur J Veterinary Med 3(3):1–6. 10.24018/ejvetmed.2023.3.3.95

[CR52] Karthik L (2022) Actinobacteria: microbiology to synthetic biology. In L. Karthik (Ed.), Actinobacteria: Microbiology to Synthetic Biology. Springer Nature. 10.1007/978-981-16-5835-8

[CR53] Kill L, Menezes E (2005) Gliricidia. In Espécies vegetais exóticas com potencialidades para o semi-árido brasileiro (pp. 301–321)

[CR55] Kneis D, Lemay-St-Denis C, Cellier-Goetghebeur S, Elena AX, Berendonk TU, Pelletier JN, Heß S (2023) Trimethoprim resistance in surface and wastewater is mediated by contrasting variants of the dfrB gene. ISME J 17(9):1455–1466. 10.1038/s41396-023-01460-7 PMC10432401 37369703

[CR108] Knights HE, Jorrin B, Haskett TL, Poole PS (2021) Deciphering bacterial mechanisms of root colonization. Environmental Microbiology Reports 13(4):428–444. 10.1111/1758-2229.12934 PMC8651005 33538402

[CR56] Li Z, Zu C, Wang C, Yang J, Yu H, Wu H (2016) Different responses of rhizosphere and non-rhizosphere soil microbial communities to consecutive Piper nigrum L. monoculture. Sci Rep 6(1):35825. 10.1038/srep35825 PMC5075767 27775000

[CR57] Liu L, Song G, Hu Y (2007) GC–MS Analysis of the Essential Oils of *Piper nigrum L.* and Piper longum L. Chromatographia 66(9–10):785–790. 10.1365/s10337-007-0408-2

[CR58] Liu M, Hong G, Li H, Bing X, Chen Y, Jing X, Gershenzon J, Lou Y, Baldwin IT, Li R (2023) Sakuranetin protects rice from brown planthopper attack by depleting its beneficial endosymbionts. Proc Natl Acad Sci U S A. 10.1073/pnas.2305007120 PMC10266023 37256931

[CR59] Lu Y, Zhou G, Ewald J, Pang Z, Shiri T, Xia J (2023) MicrobiomeAnalyst 2.0: comprehensive statistical, functional and integrative analysis of microbiome data. Nucleic Acids Res 51(W1):W310–W318. 10.1093/nar/gkad407 PMC10320150 37166960

[CR60] Luigi M (2011) Flavors and fragrances of natural and synthetic compounds 2, 2nd ed.; Wiley–Blackwell, Ed.; John Wiley & Sons Inc.: New York, NY, USA; ISBN 1118145836. [Google Scholar]

[CR61] Macedo G, van Veelen HPJ, Hernandez-Leal L, van der Maas P, Heederik D, Mevius D, Bossers A, Schmitt H (2021) Targeted metagenomics reveals inferior resilience of farm soil resistome compared to soil microbiome after manure application. Sci Total Environ 770:145399. 10.1016/j.scitotenv.2021.145399 33736375

[CR109] Maurya AP, Rajkumari J, Bhattacharjee A, Pandey P (2020) Development, spread and persistence of antibiotic resistance genes (ARGs) in the soil microbiomes through co-selection. Reviews on Environmental Health 35(4):371–378. 10.1515/reveh-2020-0035 32681784

[CR110] Meng M, Li Y, Yao H (2022) Plasmid-Mediated Transfer of Antibiotic Resistance Genes in Soil. Antibiotics 11(4):525. 10.3390/antibiotics11040525 PMC9024699 35453275

[CR62] McNally DJ, Wurms K, v, Labbé C, Bélanger RR (2003) Synthesis of C-glycosyl flavonoid phytoalexins as a site-specific response to fungal penetration in cucumber. Physiol Mol Plant Pathol 63(6):293–303. 10.1016/j.pmpp.2004.03.005

[CR65] Na X, Xu T, Li M, Zhou Z, Ma S, Wang J, He J, Jiao B, Ma F (2018) Variations of bacterial community diversity within the rhizosphere of three phylogenetically related perennial shrub plant species across environmental gradients. Front Microbiol. 10.3389/fmicb.2018.00709 PMC5915527 29720969

[CR67] Nishino K, Yamasaki S, Nakashima R, Zwama M, Hayashi-Nishino M (2021) Function and inhibitory mechanisms of multidrug efflux pumps. Front Microbiol. 10.3389/fmicb.2021.737288 PMC8678522 34925258

[CR68] Nist 11 National Institute of Standard and Technology (2025) Available online: https://webbook.nist.gov/ (accessed on 28 May

[CR69] Olanrewaju OS, Glick BR, Babalola OO (2017) Mechanisms of action of plant growth promoting bacteria. World J Microbiol Biotechnol 33(11):197. 10.1007/s11274-017-2364-9 PMC5686270 28986676

[CR72] Pignati WA, Lima FAN, deSe, Lara SSde, Correa MLM, Barbosa JR, Leão LHdaC, Pignatti MG (2017) Distribuição espacial do uso de agrotóxicos no Brasil: uma ferramenta para a Vigilância em Saúde. Ciênc saúde coletiva 22(10):3281–3293. 10.1590/1413-812320172210.17742017 29069184

[CR75] Queiroga V, Veloso N, Mendes B, Gomes JP, De Melo A, Giuliano B, Esther Ê, G., De Barros M (2023) Sistema produtivo de pimenta-do-reino (*Piper nigrum L.*). In Sistema produtivo de pimenta-do-reino (*Piper nigrum L.*). (pp. 10–126). https://ainfo.cnptia.embrapa.br/digital/bitstream/doc/1162373/1/Sistema-produtivo-pimenta-do-reino-2023.pdf

[CR76] Rad A, Astaykina A, Streletskii R, Afsharyzad Y, Etesami H, Zarei M, Balasundram SK (2022) An overview of antibiotic resistance and abiotic stresses affecting antimicrobial resistance in agricultural Soils. Int J Environ Res Public Health 19(8):4666. 10.3390/ijerph19084666 PMC9025980 35457533

[CR78] Ramakrishna W, Yadav R, Li K (2019) Plant growth promoting bacteria in agriculture: Two sides of a coin. Appl Soil Ecol 138:10–18. 10.1016/j.apsoil.2019.02.019

[CR111] Ramaroson M-L, Koutouan C, Helesbeux J-J, le Clerc V, Hamama L, Geoffriau E, Briard M (2022) Role of Phenylpropanoids and Flavonoids in Plant Resistance to Pests and Diseases. Molecules 27(23):8371. 10.3390/molecules27238371 PMC9735708 36500459

[CR79] Ranjbar R, Sami M (2017) Genetic investigation of beta-lactam associated antibiotic resistance among escherichia coli strains isolated from water sources. Open Microbiol J 11(1):203–210. 10.2174/1874285801711010203 PMC5678241 29151997

[CR112] Reimer JJ, Shaaban B, Drummen N, Sanjeev Ambady S, Genzel F, Poschet G, Wiese-Klinkenberg A, Usadel B, Wormit A (2022) Capsicum Leaves under Stress: Using Multi-Omics Analysis to Detect Abiotic Stress Network of Secondary Metabolism in Two Species. Antioxidants 11(4):671. 10.3390/antiox11040671 PMC9029244 35453356

[CR81] Rizvi A, Ahmed B, Khan MS, El-Beltagi HS, Umar S, Lee J (2022) Bioprospecting plant growth promoting rhizobacteria for enhancing the biological properties and phytochemical composition of medicinally important crops. Molecules. 10.3390/molecules27041407 PMC8880754 35209196

[CR82] Salunke P, Koche D (2023) Role of phenolic compounds in plant defense mechanism: an updated review. Indian J Appl Pure Biology v 38:1199–1215

[CR113] Schmid R, Heuckeroth S, Korf A, Smirnov A, Myers O, Dyrlund TS, Bushuiev R, Murray KJ, Hoffmann N, Lu M, Sarvepalli A, Zhang Z, Fleischauer M, Dührkop K, Wesner M, Hoogstra SJ, Rudt E, Mokshyna O, Brungs C, Pluskal T (2023) Integrative analysis of multimodal mass spectrometry data in MZmine 3. Nature Biotechnology 41(4):447–449. 10.1038/s41587-023-01690-2 PMC10496610 36859716

[CR114] Sharma A, Shahzad B, Rehman A, Bhardwaj R, Landi M, Zheng B (2019) Response of Phenylpropanoid Pathway and the Role of Polyphenols in Plants under Abiotic Stress. Molecules 24(13):2452. 10.3390/molecules24132452 PMC6651195 31277395

[CR83] Sociedade Nacional de Agricultura (2023) Brasil é o 2o maior produtor e exportador de pimenta-do-reino no mundo. Sociedade Nacional de Agricultura. https://sna.agr.br/brasil-e-o-2o-maior-produtor-e-exportador-de-pimenta-do-reino-no-mundo/

[CR84] Stanczak-Mrozek KI, Laing KG, Lindsay JA (2017) Resistance gene transfer: induction of transducing phage by sub-inhibitory concentrations of antimicrobials is not correlated to induction of lytic phage. J Antimicrob Chemother 72(6):1624–1631. 10.1093/jac/dkx056 PMC5437526 28369562

[CR115] Sun H, Wang M, Liu Y, Wu P, Yao T, Yang W, Yang Q, Yan J, Yang B (2022) Regulation of flagellar motility and biosynthesis in enterohemorrhagic Escherichia coli O157:H7. Gut Microbes 14(1):2110822. 10.1080/19490976.2022.2110822 PMC9387321 35971812

[CR85] Syiemiong D, Jha DK, Adhikari S, Mylliemngap D, Lyngdoh D, Warlarpih JP, Paul N, Lamare KM, Wahlang C, Lyngkhoi R (2022) Rhizospheres of Rubus ellipticus and Ageratina riparia from Meghalaya exhibit Actinomycetota that promote plant growth. J Appl Biology Biotechnol. 10.7324/JABB.2023.110210

[CR87] Thapa BB, Huo C, Budhathoki R, Chaudhary P, Joshi S, Poudel PB, Magar RT, Parajuli N, Kim KH, Sohng JK (2024) Metabolic comparison and molecular networking of antimicrobials in streptomyces species. Int J Mol Sci 25(8):4193. 10.3390/ijms25084193 PMC11050201 38673777

[CR88] Thongphichai W, Pongkittiphan V, Laorpaksa A, Wiwatcharakornkul W, Sukrong S (2023) Antimicrobial activity against foodborne pathogens and antioxidant activity of plant leaves traditionally used as food packaging. Foods 12(12):2409. 10.3390/foods12122409 PMC10297150 37372620

[CR91] Trindade R, Almeida L, Xavier L, Andrade EH, Maia JG, Mello A, Setzer WN, Ramos A, Silva JK R (2021) Influence on secondary metabolism of *Piper nigrum* L. by co-inoculation with arbuscular mycorrhizal fungi and fusarium solani f. sp. piperis. Microorganisms 9(3):484. 10.3390/microorganisms9030484 PMC7996571 33669088

[CR116] Ullah S, Hussain B, Iqbal N, Raza MM, Salam M, Vasudhevan P, Sani SGAS, Pu S (2025) Microbial ecosystem disruption under persistent organic pollutant stress: Consequences for soil biogeochemistry and environmental sustainability – A review. Environmental Chemistry and Ecotoxicology 7:2643–2659. 10.1016/j.enceco.2025.10.017

[CR92] van den Dool H, Dec. Kratz P (1963) A generalization of the retention index system including linear temperature programmed gas—liquid partition chromatography. J Chromatogr A 11:463–471. 10.1016/S0021-9673(01)80947-X 14062605

[CR93] Wang M, Carver JJ, Phelan Vv, Sanchez LM, Garg N, Peng Y, Nguyen DD, Watrous J, Kapono CA, Luzzatto-Knaan T, Porto C, Bouslimani A, Melnik A, v, Meehan MJ, Liu W-T, Crüsemann M, Boudreau PD, Esquenazi E, Sandoval-Calderón M, Bandeira N (2016) Sharing and community curation of mass spectrometry data with Global Natural Products Social Molecular Networking. Nat Biotechnol 34(8):828–837. 10.1038/nbt.3597 PMC5321674 27504778

[CR94] Wang Z, Solanki MK, Yu Z-X, Anas M, Dong D-F, Xing Y-X, Malviya MK, Pang F, Li Y-R (2021) Genome characteristics reveal the biocontrol potential of actinobacteria isolated from sugarcane rhizosphere. Front Microbiol. 10.3389/fmicb.2021.797889 PMC8740303 35003029

[CR95] Wang Z, Lu K, Liu X, Zhu Y, Liu C (2023) Comparative functional genome analysis reveals the habitat adaptation and biocontrol characteristics of plant growth-promoting bacteria in NCBI databases. Microbiol Spectr. 10.1128/spectrum.05007-22 PMC10269705 37098923

[CR96] Wood DE, Lu J, Langmead B (2019) Improved metagenomic analysis with Kraken 2. Genome Biol 20(1):257. 10.1186/s13059-019-1891-0 PMC6883579 31779668

[CR97] Xiao Y, Lu H, Liu Y, Sang Y, Sun J (2022) Whole-genome sequencing and functional analysis of a novel chitin-degrading strain Rhodococcus sp. 11 – 3. J Biosci Bioeng 134(2):167–173. 10.1016/j.jbiosc.2022.04.010 35644796

[CR99] Yasir M, Khan R, Ullah R, Bibi F, Khan I, Mustafa Karim A, Al-Ghamdi AK, Azhar EI (2022) Bacterial diversity and the antimicrobial resistome in the southwestern highlands of Saudi Arabia. Saudi J Biol Sci 29(4):2138–2147. 10.1016/j.sjbs.2021.11.047 PMC9072880 35531257

[CR117] York LM, Carminati A, Mooney SJ, Ritz K, Bennett MJ (2016) The holistic rhizosphere: integrating zones, processes, and semantics in the soil influenced by roots. Journal of Experimental Botany 67(12):3629–3643. 10.1093/jxb/erw108 26980751

[CR100] Yuan K, Yu K, Yang R, Zhang Q, Yang Y, Chen E, Lin L, Luan T, Chen W, Chen B (2019) Metagenomic characterization of antibiotic resistance genes in Antarctic soils. Ecotoxicol Environ Saf 176:300–308. 10.1016/j.ecoenv.2019.03.099 30947033

[CR101] Zachariah TJ, Safeer AL, Jayarajan K, Leela NK, Vipin TM, Saji KV, Shiva KN, Parthasarathy VA, Mammootty KP (2010) Correlation of metabolites in the leaf and berries of selected black pepper varieties. Sci Hort 123(3):418–422. 10.1016/j.scienta.2009.09.017

[CR102] Zhang R, Xia H, Guo P, Qin Z (2008) Variation in the replication loci of Streptomyces linear plasmids. FEMS Microbiol Lett 290(2):209–216. 10.1111/j.1574-6968.2008.01432.x 19054078

[CR104] Zheng D, Yin G, Liu M, Hou L, Yang Y, van Boeckel TP, Zheng Y, Li Y (2022) Global biogeography and projection of soil antibiotic resistance genes. Sci Adv. 10.1126/sciadv.abq8015 PMC9668297 36383677

[CR105] Zhong L, Lin Y, Wang C, Niu B, Xu Y, Zhao G, Zhao J (2022) Chemical profile, antimicrobial and antioxidant activity assessment of the crude extract and its main flavonoids from tartary buckwheat sprouts. Molecules 27(2):374. 10.3390/molecules27020374 PMC8779668 35056695

