# Supplementary material for: Influence of Gliricidia sepium living support on rhizosphere bacterial recruitment and fruit metabolomics in black pepper (Piper nigrum)
Source: World J Microbiol Biotechnol. 2026 Oct 31;42(11):636. doi: 10.1007/s11274-026-05278-1 (PMC13722817; doi:10.1007/s11274-026-05278-1)
Supplement: Supplementary file 1 [file 11274_2026_5278_MOESM1_ESM.docx]

**SUPPLEMENTARY MATERIAL**

**Supplementary Table I - PERMANOVA analysis**

|  | **Df** | **SumOfSqs** | **R2** | **F** | **Pr(>F)** |
| --- | --- | --- | --- | --- | --- |
| SampleType | 1 | 0.03358685295 | 0.5951575058 | 14.07821899 | 0.0017 |
| Support | 1 | 0.002213501902 | 0.03922315297 | 0.9278084067 | 0.3669 |
| SampleType:Support | 1 | 0.001547345274 | 0.02741888783 | 0.6485831123 | 0.499 |
| Residual | 8 | 0.01908585338 | 0.3382004534 | - | - |
| Total | 11 | 0.05643355351 | 1 | - | - |

**Supplementary Table II - PERMANOVA pairwise analysis**

| Comparison | R2 | F | P | P_adjusted |
| --- | --- | --- | --- | --- |
| RL vs RD | 0.1147237511 | 0.518363624 | 1 | 1 |
| RL vs SL | 0.7007256132 | 9.365661001 | 0.1 | 0.6 |
| RL vs SD | 0.7116429043 | 9.871689163 | 0.1 | 0.6 |
| RD vs SL | 0.5688398002 | 5.277294153 | 0.1 | 0.6 |
| RD vs SD | 0.6207283897 | 6.546531539 | 0.1 | 0.6 |
| SL vs SD | 0.2237673913 | 1.153094517 | 0.4 | 0.8 |

**Supplementary Table III - Bacterial taxon recruited by RL**

| **Actinomycetota** | **RD** | **RL** | **SD** | **SL** |
| --- | --- | --- | --- | --- |
| Actinoplanes sichuanensis | Absent | Present | Present | Present |
| Actinoplanes sp. OR16 | Absent | Present | Present | Present |
| Arthrobacter sp. J3.49 | Absent | Present | Present | Present |
| Aurantimicrobium minutum | Absent | Present | Present | Present |
| Aurantimicrobium sp. INA4 | Absent | Present | Present | Present |
| Brevibacterium sp. Ap13 | Absent | Present | Present | Present |
| Candidatus Planktophila vernalis | Absent | Present | Present | Present |
| Gordonia ajococcus | Absent | Present | Present | Present |
| Microbacterium sp. AISO3 | Absent | Present | Present | Present |
| Microbacterium sp. Se5.02b | Absent | Present | Present | Present |
| Microbacterium sp. Se63.02b | Absent | Present | Present | Present |
| Mycobacterium orygis | Absent | Present | Present | Present |
| Paenarthrobacter sp. R1 | Absent | Present | Present | Present |
| Pseudonocardia sp. EC080619-01 | Absent | Present | Present | Present |
| Rhodococcus sp. 11-3 | Absent | Present | Present | Present |
| Streptomyces griseorubiginosus | Absent | Present | Present | Present |
| Streptomyces sp. ICC1 | Absent | Present | Present | Present |
| Streptomyces sp. LBUM 1475 | Absent | Present | Present | Present |

**Supplementary Table IV - Bacterial taxon recruited by RD**

| **Actinomycetota** | **RD** | **RL** | **SD** | **SL** |
| --- | --- | --- | --- | --- |
| Arthrobacter sp. AQ5-05 | Present | Absent | Present | Present |
| Arthrobacter sp. FW306-05-C | Present | Absent | Present | Present |
| Arthrobacter sp. H16F315 | Present | Absent | Present | Present |
| Candidatus Planktophila sulfonica | Present | Absent | Present | Present |
| Corynebacterium caspium | Present | Absent | Present | Present |
| Corynebacterium sp. sy039 | Present | Absent | Present | Present |
| Dactylosporangium fulvum | Present | Absent | Present | Present |
| Microbacterium sp. PAMC21962 | Present | Absent | Present | Present |
| Micrococcus sp. V7 | Present | Absent | Present | Present |
| Mycobacterium conspicuum | Present | Absent | Present | Present |
| Mycolicibacterium sp. P1-18 | Present | Absent | Present | Present |
| Nocardioides sp. LMS-CY | Present | Absent | Present | Present |
| Rhodococcus sp. AD45 | Present | Absent | Present | Present |
| Rhodococcus sp. AH-ZY2 | Present | Absent | Present | Present |
| Rhodoluna sp. KAS3 | Present | Absent | Present | Present |
| Streptomyces sp. 14R-10 | Present | Absent | Present | Present |
| Streptomyces sp. S501 | Present | Absent | Present | Present |
| Streptomyces sp. Tue6028 | Present | Absent | Present | Present |

**Supplementary Table V - Resistance Mechanism identifies and number of classes of drug resistance.**

| **ARO Term** | **Mechanism** | **Family Gene** | **Nº Dclass** |
| --- | --- | --- | --- |
| TriB | Efflux pump | resistance-nodulation-cell division (RND) | 1 |
| TriC | Efflux pump | resistance-nodulation-cell division (RND) | 1 |
| YajC | Efflux pump | resistance-nodulation-cell division (RND) | 10 |
| acrB | Efflux pump | resistance-nodulation-cell division (RND) | 7 |
| amrB | Efflux pump | resistance-nodulation-cell division (RND) | 1 |
| mdtB | Efflux pump | resistance-nodulation-cell division (RND) | 1 |
| mexM | Efflux pump | resistance-nodulation-cell division (RND) | 1 |
| opcM | Efflux pump | resistance-nodulation-cell division (RND) | 2 |
| rsmA | Efflux pump | resistance-nodulation-cell division (RND) | 3 |
| smeS | Efflux pump | resistance-nodulation-cell division (RND) | 4 |
| MexC | Efflux pump | resistance-nodulation-cell division (RND) | 9 |
| MexD | Efflux pump | resistance-nodulation-cell division (RND) | 9 |
| OprZ | Efflux pump | resistance-nodulation-cell division (RND) | 4 |
| Pseudomonas aeruginosa CpxR | Efflux pump | resistance-nodulation-cell division (RND) | 15 |
| lfrA | Efflux pump | major facilitator superfamily (MFS) | 1 |
| rosA | Efflux pump | major facilitator superfamily (MFS) | 1 |
| qacG | Efflux pump | small multidrug resistance (SMR) | 1 |
| qacJ | Efflux pump | small multidrug resistance (SMR) | 1 |
| Klebsiella pneumoniae KpnF | Efflux pump | small multidrug resistance (SMR) | 7 |
| BKC-1 | Antibiotic inactivation | BKC Beta-lactamase | 1 |
| CTX-M-11 | Antibiotic inactivation | CTX-M beta-lactamase | 1 |
| KPC-22 | Antibiotic inactivation | KPC beta-lactamase | 4 |
| SHV-160 | Antibiotic inactivation | SHV beta-lactamase | 3 |
| TEM-230 | Antibiotic inactivation | TEM beta-lactamase | 4 |
| AAC(3)-IIb | Antibiotic inactivation | AAC(3) | 1 |
| mphA | Antibiotic inactivation | macrolide phosphotransferase (MPH) | 1 |
| ramA | Antibiotic inactivation | rifampin ADP-ribosyltransferase (Arr) | 1 |
| vanR gene in vanO cluster | Target alteration | glycopeptide resistance gene cluster; vanR | 1 |
| dfrB3 | Replacement target | trimethoprim resistant dihydrofolate reductase dfr | 1 |

**Supplementary Table VI - Genes related to antimicrobial resistance are unique to each sample.**

| **RD** | **RL** | **SD** | **SL** |
| --- | --- | --- | --- |
| CTX-M-11 | mdtB | AAC(3)-IIb | MexC |
| Klebsiella pneumoniae KpnF | qacG | BKC-1 | OprZ |
| SHV-160 | - | KPC-22 | TriC |
| qacJ | - | MexD | YajC |
| - | - | Pseudomonas aeruginosa CpxR | amrB |
| - | - | lfrA | mphA |
| - | - | rosA | rsmA |
| - | - | smeS | - |
