# Supplementary material for: Influence of Gliricidia sepium living support on rhizosphere bacterial recruitment and fruit metabolomics in black pepper (Piper nigrum)
Source: World J Microbiol Biotechnol. 2026 Oct 31;42(11):636. doi: 10.1007/s11274-026-05278-1 (PMC13722817; doi:10.1007/s11274-026-05278-1)
Supplement: Supplementary file 2 [file 11274_2026_5278_MOESM2_ESM.docx]

**SUPPLEMENTARY MATERIAL**

**Supplementary Figure S1 – Full Uncropped Agarose Gel Electrophoresis Images**
